# Trust and vaccine hesitancy during the COVID-19 pandemic: A cross-national analysis

**DOI:** 10.1016/j.jvacx.2023.100299

**Published:** 2023-04-06

**Authors:** Will Jennings, Viktor Valgarðsson, Lawrence McKay, Gerry Stoker, Eduardo Mello, Hasan Muhammad Baniamin

**Affiliations:** a University of Southampton, UK; b Fundação Getulio Vargas, Brazil; c North South University, Bangladesh

**Keywords:** COVID-19, Vaccination, Trust, Misinformation

## Abstract

Previous studies of vaccine hesitancy in the context of COVID-19 have reported mixed results in terms of the role played by political and institutional trust. This study addresses this ambiguity with a global analysis of the relationship between trust and vaccine hesitancy, disentangling the effects of generalized trust orientations, trust in specific institutions and conspiracy mentality. It first draws upon a cross-national survey of 113 countries to demonstrate that trust in government is a predictor of vaccine hesitancy across global regions. It further draws on original surveys fielded in seven countries (France, Germany, Spain, Argentina, Croatia, Brazil, India), which deploy a diverse range of measures, to disentangle the individual-level predictors of vaccine hesitancy. Our findings confirm the robust effects of trust in government across countries, but when including other trust measures in the same models, the most robust effects are those of trust in health institutions and conspiracy mentality. Weaker associations are observed for right-wing ideology and online political engagement, while the consumption of traditional media tends to predict the willingness of individuals to be vaccinated.

## Highlights

• Political trust is widely believed to play a critical role in shaping vaccine acceptance;
• Empirical studies have reported mixed results due to the wide range of ways that trust is measured and operationalised;
• Global analysis of survey data from 113 countries shows trust in government is a strong predictor of vaccine hesitancy.
• Cross-national analysis from seven countries shows that when trust measures are disaggregated, the most robust effects relate to trust in health institutions and conspiracy mentality.

The global battle against COVID-19 has drawn attention to the perceived critical importance of political trust for public health interventions designed to mitigate the spread of infectious disease [[3], [4], [8], [9]]. This perception seems well-founded: citizens’ trust in authorities and social institutions speaks to whether they believe that they are actually looking out for their interests and has thus long been understood as fundamental to cooperation between government and the public [[10], [13]]. Indeed, trust has been found to be importantly related to countries’ general success in combating the pandemic on the aggregate level [[3], [30]] and with citizens’ compliance with public health guidelines on the individual level [[14], [26], [27], [29]].

However, when it comes to the particular topic of the role of political trust in shaping vaccine acceptance, studies have reported mixed results. There appears to be no doubt that some kind of trust matters, but while Rozek et al. [21] report in their 17-country study that trust in *health* institutions matter, they report no effects for trust in *political* leaders. Similarly, Kerr et al. [14] find in their 25-sample study that trust in *experts* plays an important role, but they report non-significant effects for trust in *government* in all but one of their samples. On the other hand, Lazarus et al. [16] find a significant effect for trust in government in their 19-country study and Lindholt et al. [17] find in their 8-country study that trust in government has an effect in addition to trust in scientists and health authorities, although that effect is only marginally significant in their full model. Finally, Allington et al. [1] find in their study of the UK that trust in government had a significant effect but a much smaller one than trust in scientists and medical professionals. Other studies on the topic have been limited by their use of convenience samples, although these tend to find significant effects for political trust [[16], [22], [25]].

In summary, of the four large, cross-national studies published to date on the topic, two report significant effects for political trust on vaccine acceptance, whereas two report no significant effects. In this study, we directly address this ambiguity in the existing literature, using secondary survey data from over 100 countries and original survey data from seven countries, with the latter explicitly designed to disentangle the role of different types of trust in seven countries. The broad concept of political trust is typically not measured in a systematic or theoretically driven way in prior studies; instead, different measures of trust in various entities tend to be lumped together in statistical models, without great clarity about the reasons behind the choice of variables, the correlations between them or how the measures overlap. In this study, we move beyond these previous approaches by distinguishing between generalized trust orientations and more specific confidence in particular institutions, by distinguishing between political *trust*, *mistrust*, *distrust* and *conspiracy mentality* as distinct types of attitudes within the “trust” family, and by explicitly interrogating the inter-relationships between different types of trust and vaccine acceptance. Finally, our original surveys contain a broader range of relevant measures of personal, attitudinal and behavioural factors than most others, allowing us to compare the effects of trust with factors such as media use, personality traits and demographics to a greater extent than prior studies.

Our approach in this paper thus seeks to advance our understanding of the role(s) of trust in vaccine acceptance by addressing these limitations of prior studies. Firstly, we undertake a global analysis of the degree to which trust predicts vaccine hesitancy, based on high quality survey data from over 100 countries. This enables us to determine how general the link between trust and vaccine hesitancy is, covering a broader scope of countries than any prior study on the individual level. Secondly, we report results from our original surveys conducted in seven countries, which included an extensive set of measures which allow us to disentangle the relationship between different types of trust and vaccine acceptance and compare with the role of related factors such as personality traits, cognition and political ideology. In terms of trust, we include measures of generalized political *trust*, *mistrust* and *distrust*, reflecting insights from recent studies that suggest that lack of trust may either denote a type of cautious scepticism (mistrust) or settled dissatisfaction (distrust) – and that the two may have different implications for vaccine hesitancy, in that we would expect distrust but not necessarily mistrust to drive it ([2]; [28]; [12]). In addition, we include a scale measuring ‘conspiracy mentality’ (developed by 5 and more conventional measures of trust in representative institutions, health institutions and the media as well as a measure of *social* trust, separating these in our analysis and comparing their relative effects. This offers a more comprehensive test of the precise ways that trust influences behaviour and its relative importance.

Our empirical findings confirm that political trust is a consistent predictor of vaccine hesitancy across the world, one that is robust to the inclusion of multiple controls and holds in over 90 percent of the more than 100 countries covered here. In our more detailed analysis of the role of trust, using original survey data, we find that generalised distrust in government is positively related to vaccine hesitancy and generalised trust is negatively related to it, whereas the effects of political mistrust and social trust are non-significant, but conspiracy mentality has the strongest effect by some margin. When separating political trust into trust in different institutions, we find, in line with previous studies, that trust in health institutions stands out as by far the most robust, but this is fairly strongly related to confidence in political institutions, which has a significant effect when the former is not included in the same model. Indeed, trust in health institutions, conspiracy mentality and age are the only predictors whose significant effects persist even after including all 29 variables in our analysis in the same model. Consuming news in traditional media is also a strong (negative) predictor of hesitancy and those who identify on the right in politics are more likely to be hesitant, whereas the effects of other factors are weaker and less consistent. By combining breadth and depth of analysis, we highlight the consistency of trust as a predictor of vaccine hesitancy and shed light on the dynamic role that different types of trust play, with conspiracy mentality and trust in health institutions standing out as the most potent predictors.

## Methods

### Wellcome global monitor data

For the global analysis, we use data from The Wellcome Global Monitor (WGM) included in the Gallup World Poll. Between October 2020 and January 2021 inclusive, telephone surveys were completed by nearly 120,000 respondents in 113 countries, which collectively represent 90 % of the global population aged 15 +. The surveys concentrated on themes related to COVID-19, including its economic impacts, and crucially featured questions on both vaccine uptake intentions and on trust. The measures used here asked respondents: (a) how much they trusted the national government in their country, and (b) whether they would agree to be vaccinated if a vaccine to prevent coronavirus “was available right now at no cost”.^1^

The WGM also included demographic information on respondents’ age, sex, education, income and employment status. In terms of sample size, 98,166 respondents in 105 countries are valid on the vaccine and trust measures, with 94,620 in 102 countries valid once control variables are added in a regression model.

### Original survey data

For the second part of the analysis, we use data from online nationally representative surveys in seven major countries – France, Germany, Spain, Croatia, Argentina, Brazil and India – which we conducted between September and December 2021. Each of our surveys apply quotas on multiple demographic factors, which include age, gender, and region for all surveys (and additionally social class for NetQuest surveys and education and past vote for YouGov surveys). Full details of the survey fieldwork, sample size, survey company and mode/design are reported in Table 1.

**Table 1:** Original survey details.

**Country**	**Fieldwork**	**Sample**	**Company**	**Design**	Vaccination rate*
France	23.09.2021 – 20.11.2021	1,548	YouGov	Online, nationally representative	65 % (75 %)
Germany	14.10.2021 – 23.11.2021	1,558	YouGov	Online, nationally representative	65 % (70 %)
Spain	23.09.2021 – 26.09.2021	1,022	YouGov	Online, nationally representative	77 % (79 %)
Croatia	29.09.2021 – 06.10.2021	1,017	YouGov	Online, nationally representative	40 % (46 %)
Argentina	29.09.2021 – 08.10.2021	1,113	YouGov	Online, nationally representative	50 % (66 %)
Brazil	06.12.2021 – 28.12.2021	2,123	NetQuest	Online, nationally representative	64 % (77 %)
India	22.09.2021 – 30.09.2021	1,040	NetQuest	Online, nationally representative (of the literate population)	15 % (44 %)
*Total*	*22.09.2021 – 28.11.2021*	*9,421*			

*Note:* France includes a top-up sample of 498 respondents 19.11.2021–20.11.2021; Germany includes top-up sample of 534 respondents 19.11.2021–20.11.2021. *Share of people vaccinated against COVID-19 at start of survey fieldwork: complete initial vaccine protocol (partially vaccinated).

The dependent variable is *vaccine willingness*, the inverse of vaccine hesitancy. At the time of fieldwork, the distribution of free vaccines was becoming a reality rather than (as with the data in our previous analysis) a theoretical future possibility, even in countries where the rate of doses had been stalled by poor availability (such as in the case of India, which may be reflected in its low level of first doses and full vaccinations received), so the measures reflect actual reported prior behaviour, rather than reported vaccine *intentions*. In the survey, we asked respondents the following pair of questions, coding as vaccine ‘willing’ (1) those respondents who answered that they had received a dose or that they would if offered. Those who had declined the dose offered, or would not get a future dose if offered, are coded as unwilling (0).^2^ The questions were formulated as follows:

*Q13. Which of these applies to you?*
  (1) *I have received one or two doses of a COVID-19 vaccine*
  (2) *I have been offered a vaccine against COVID-19, but declined the offer to be vaccinated*
  (3) *I have not yet been offered a vaccine against COVID-19*
  (4) *Prefer not to say*
*Q14. To what extent, if at all, do you agree, or disagree, with the following statements?*
  *If I was offered a vaccine for COVID-19, I would get it [ASK IF Q13 = 3]*
  (1) *Strongly agree*
  (2) *Tend to agree*
  (3) *Neither agree nor disagree*
  (4) *Tend to disagree*
  (5) *Strongly disagree*
  (6) *Don’t know*

Turning to our independent variables, the survey includes multiple measures of trust. First, it includes conventional measures of confidence in institutions, drawn from the *World Values Survey*, from which we derive three separate measures of institutional trust: 1) trust in health institutions, which is calculated for each respondent as the mean of trust in the World Health Organisation (WHO) and “the health service in your country”,^3^ 2) trust in the media, which is the mean of trust in “the press” and “TV”, and 3) trust in political institutions, which is calculated as the mean of trust in “parliament”, “political parties” and “the government in your country”. We also include a module developed in previous studies to measure generalized orientations of trust, mistrust and distrust towards government [[7], [14]]. The following set of questions was asked in all seven countries:

*To what extent do you agree, or disagree, with the following statements?*
  (1) *The government is honest and truthful*
  (2) *In general, the government usually does the right thing*
  (3) *The government usually has good intentions*
  (4) *I am usually cautious about trusting the government*
  (5) *I am unsure whether to believe the government*
  (6) *The government acts unfairly towards people like me*
  (7) *The government usually ignores my community*
  (8) *The government doesn’t respect people like me*
    (1) *Strongly agree*
    (2) *Tend to agree*
    (3) *Neither agree nor disagree*
    (4) *Tend to disagree*
    (5) *Strongly disagree*
    (6) *Don’t know*

Following previous work, we took the first three statements (1–3) to indicate trust, the next two (4–5) to reflect mistrust, and the last three (6–8) to measure distrust. For each set of statements, we created scales for each respondent taking the mean level of agreement with the statements (on the Likert scale) before rescaling the values to 0–1. Cronbach’s alpha scores vary between 0.60 (mistrust) to 0.84 (distrust) and 0.90 (trust), all of which pass the 0.6 threshold established as acceptable by Nunally and Bernstein [20].

Turning to our other independent variables, we provide more detail in Online Appendix B, and only summarise our measures briefly here. In terms of demographics, we include age, gender, and a dummy for university level education. Our models include left–right ideological self-placement, measured on a 0–10 scale. We further incorporate measures of the ‘Big Five’ personality traits, based on the ‘Short 15-item Big Five Inventory (BFI-S)’ devised and validated by Lang et al. [15], creating scales for the mean (dis)agreement with the statements measuring each of the Big Five. We also include a scale of ‘conspiracy mentality’, designed for use across cultures and languages, based on the 5-item scale by Bruder et al. [5], albeit with a slightly modified question and response scale. We take the simple mean of these items as our measure of conspiracy mentality. Finally, we include a measure of cognitive reflection based on Frederick (2005; see also [25]), which principally measures the ability or disposition of respondents to reflect on a question and resist reporting the first response that comes to mind (using classic games/experiments from behavioural economics). We take the sum of correct answers (0–3) to be the respondent’s cognitive reflection score. Finally, we include measures of media consumption and activity: how much respondents follow news in different types of media and whether they have recently ‘fact-checked’ articles or posted political content online. All these scales and all other variables used in our analysis are rescaled to 0–1 to enable direct comparison of effects. After removing all cases with missingness in the final model, 6,210 respondents are retained for the multivariate analysis.

## Results

### Global analysis: Wellcome Global Monitor data

**Fig. 1:**
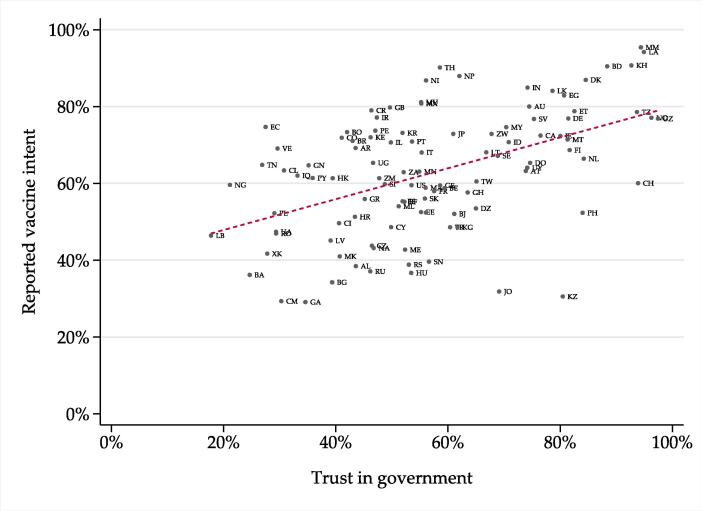
Trust in government vs self-reported vaccine intentions (aggregate-level), Wellcome Global Monitor. Countries denoted by Alpha-2 ISO codes, see: https://www.iban.com/country-codes.

We begin with an analysis of the relationship between political trust and vaccine hesitancy across the globe. Is this robust, and does it consistently emerge globally? To answer this, we turn to the Wellcome Global Monitor included in the Gallup World Poll, which includes measures of trust in government and willingness to be vaccinated, along with demographic measures. Further information about this data source and the variables used is presented in the methods section below and in Online Appendix B. We first present the country-level aggregate relationships between trust in government and vaccine intention on the country-level (Fig. 1).^4^ The y-axis represents the percentage of respondents in each country who said that they would agree to be vaccinated against the coronavirus if a vaccine was available at no cost,^5^ while the x-axis represents the percentage of respondents who said they trusted the national government in their country ‘a lot’ or had ‘some’ trust in it.^6^ The aggregate correlation between these two variables is positive and moderate (0.49, p < 0.001). The simple bivariate regression line shows that at the lowest observed levels of trust (20 %), fewer than 50 % are expected to take vaccines, while at the highest levels (near to 100 %), nearly 80 % would accept a no-cost vaccine.

**Fig. 2:**
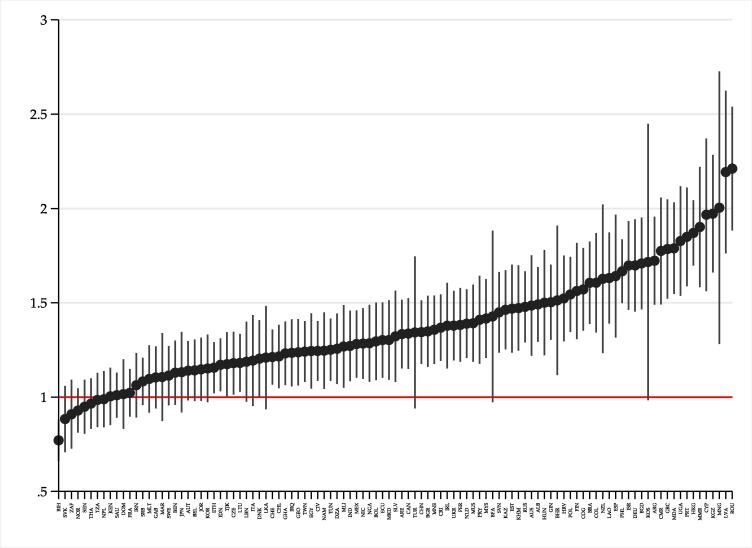
Bivariate regression of trust in government on self-reported vaccine willingness, odds ratios (individual-level, by country). Countries denoted by Alpha-3 ISO codes, see: https://www.iban.com/country-codes.

To explore how far this relationship is universal, we run bivariate logistic regression models of vaccine intention on trust within each country on the individual level and present the odds ratios for each country in Fig. 2 (where trust in government is an ordinal variable that takes the values 1 ‘not at all’, 2 ‘not much’, 3 ‘some’ and 4 ‘a lot’). While there is significant cross-national heterogeneity, it is striking that there is statistically significant (at the 95 per cent confidence level) positive relationship between trust in government and vaccine willingness in 75 out of 105 countries. In just a single country (Bosnia Herzegovina), the relationship between trust and vaccine willingness is significantly negative (i.e. confidence intervals of the estimated odds ratio do not cross at 1.0). In another 29 countries, the relationship was not statistically significant, interestingly including both high trust countries, such as Norway and Sweden, and low trust countries, such as Slovakia, Senegal and Serbia. The average odds ratio across countries is equal to 1.4, meaning that each one unit increase in the ordinal scale of trust in government typically leads to a 40 % increase in the likelihood of being willing to be vaccinated.

**Fig. 3:**
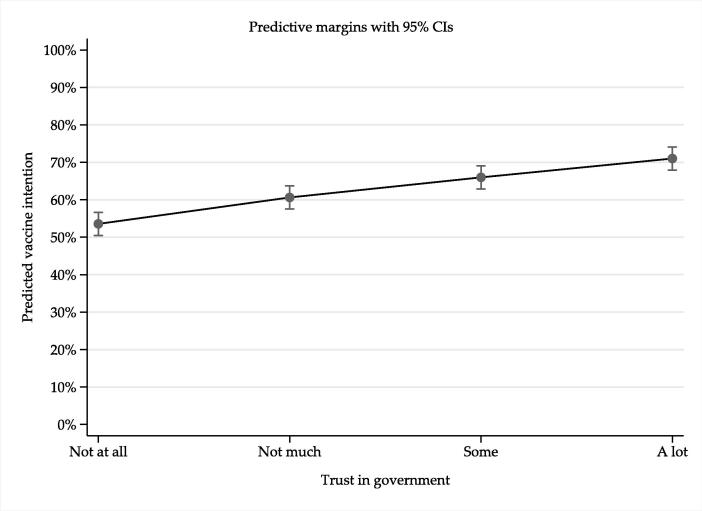
Marginal predictions of vaccine intention by levels of trust in government in the 2020 Wellcome Global Monitor survey, from multi-level regression models controlling for individuals’ demographics and countries’ HDI.

Turning to a more sophisticated analysis of this relationship, we next run multi-level regression models on the entire dataset (with country as the second level). These again predict vaccine intent as a function of trust in government,^7^ and add several individual-level controls: age (in four categories), sex, education (a binary variable where those with university education are coded as equal to 1), employment status and income on the individual level (in quintiles). At the country-level, we include the Human Development Index (HDI) score from the UN Human Development Reports (to partly account for the potential confounding effect of societal welfare levels, which may drive both trust and vaccine intention). The full results from these regression models are presented in Online Appendix, Table A1, and they show that moving through each stage from the lowest to the highest level of trust in government is associated with a significant increase in vaccine willingness, when controlling for individual and contextual factors. Fig. 3 presents the marginal effects of each category of trust from these models, illustrating that the global prediction (holding all control variables constant) for vaccine intention is about 54 % for those who say they trust their national government “not at all”, whereas it is about 71 % for those who say they trust it “a lot”. Thus, the global relationship between political trust and vaccine intention appears to be significant and substantial even when accounting for demographic and contextual factors.

## Detailed analysis: original surveys in seven countries

While the Wellcome Global Monitor provides impressive global scope in terms of the total number of countries and proportion of the global population covered, it is understandably limited in how far it explores alternative measures of trust, as well as other relevant values, beliefs and behaviours which may account for or overshadow the relationship. We build upon this through fielding our own bespoke survey that specifically explored multiple dimensions of trust and a range of related factors in multiple countries. For our study, we are therefore able to draw on an array of measures that distinguish between categories of generalized *trust*, *mistrust* and *distrust* in government, as well as more conventional measures of trust in specific institutions, where we differentiate between political institutions (parliament, political parties and the national government), health institutions (the national health service and WHO) and the media (the press and TV). We also include various measures of conspiratorial mentality, political ideology, cognitive reflection, personality traits and traditional and online media use, as well as demographic information. This approach enables us to explore the degree to which a much wider set of measures of political attitudes are predictive of vaccine willingness/hesitancy, and to situate different types of trust in that context. We provide further details on these data and measures in the methods section and the full questionnaire in Online Appendix A.

**Fig. 4:**
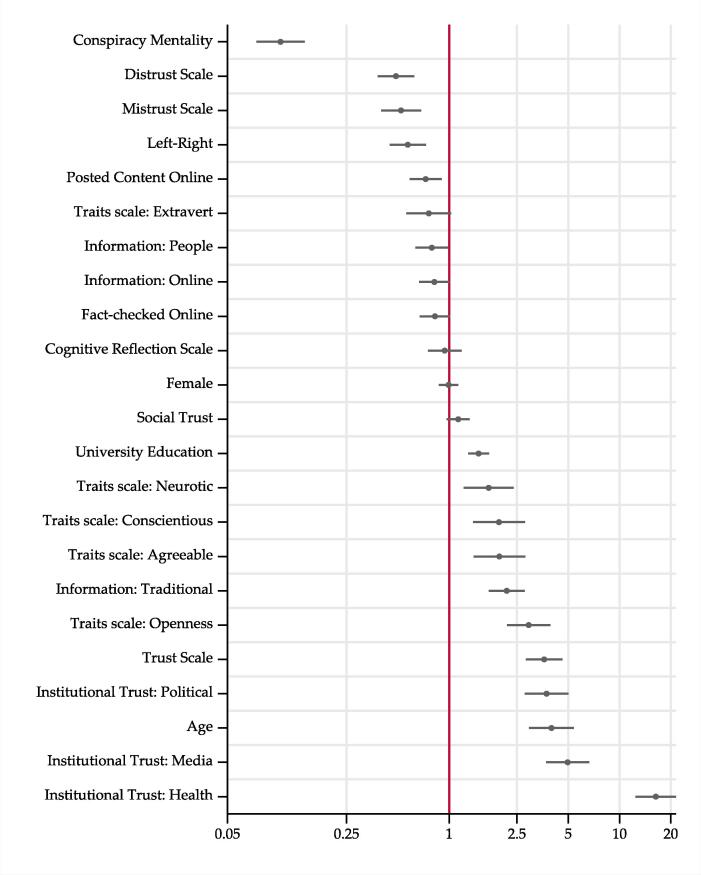
Bivariate logistic regression of vaccine willingness, odds ratios.

We begin by considering the degree to which each of our measures in isolation is associated with willingness to be vaccinated against COVID-19. Bivariate logistic regression models are calculated for all predictors for the full, pooled seven-country sample, with the odds ratios plotted in Fig. 4. In the figure, the dark circles indicate the coefficient value, while the whiskers indicate the 95 per cent confidence intervals. The red line indicates the point at which there is no significant relationship between the variables if those confidence intervals cross.

Several measures of trust have statistically significant bivariate relationships with vaccine willingness. The strongest positive relationship is observed for trust in health institutions (odds ratio = 16.32, 95 % CI 12.40 to 21.48): in other words, those most trusting were between 12 and 21 times more likely to accept the vaccine than those lowest in trust. Trust in the media (OR 4.96, 95 % CI 3.70 to 6.64) and political institutions (OR 3.72, 95 % CI 2.78 to 5.01) also have significant, but weaker, bivariate relationships with vaccination willingness.

As expected, generalized trust orientations towards government also have a positive effect on willingness (OR: 3.61, 95 % CI 2.83 to 4.62), whereas distrusting and mistrusting orientations have negative effects with mistrusters (OR: 0.49, 95 % CI 0.38 to 0.62) and distrusters (OR: 0.52, 95 % CI 0.40 to 0.68) each about half as likely. Perhaps surprisingly, the difference between the effects of distrust and mistrust are small, and social trust has weak and non-significant effects. Besides trust, we observe numerous other significant relationships. Most notably, those with most ‘conspiracy mentality’ are about 10 % as likely as the least conspiratorial to be willing to be vaccinated (OR = 0.10, 95 % CI 0.07 to 0.14) and the oldest respondents are four times as likely as the youngest (OR = 3.98, 95 % CI 2.94 to 5.40). Engagement with different information sources also appears related to vaccine hesitancy: online information consumption, sharing and fact-checking material online are associated with slightly lower willingness to get vaccinated, though the positive association with consumption of *traditional* media is slightly larger in magnitude. People who identify as on the right of the political spectrum are also somewhat less likely to get vaccinated, as are those who score higher on some of the Big Five personality trait scales; especially on ‘openness’, whereas cognitive reflection shows no association. Finally, as in other studies, university education is related to greater willingness to get vaccinated for COVID-19.

While informative in a descriptive sense, these bivariate relationships may well be confounded by various related demographic and attitudinal factors. To get closer to a causal relationship, we next run a series of multivariate regression models, to isolate and disentangle the effects of specific factors. We estimate six separate ‘block’ models of related variables here, each retaining a selection of closely related variables. Each of the block models controls for demographic factors (gender, age, education), left–right ideology and country dummies (because the dependent and independent variables are likely to vary by country and the other control variables, in ways which might result in spurious effects reported in the absence of these controls). In Online Appendix A, we also report results of a single model including all 29 variables. We focus on block models in this discussion, because including so many correlated variables in one model creates problems of multicollinearity, wide confidence intervals and very unintuitive interpretations of individual coefficients 29. We see this model primarily as a conservative test of which predictors are the most robust of all: these turn out to be trust in health institutions, conspiracy mentality, age, and consumption of traditional media. However, we run all our block models on the same sample of respondents with non-missing values on all variables (N = 6,235), to enable comparison of effects between models.

**Fig. 5:**
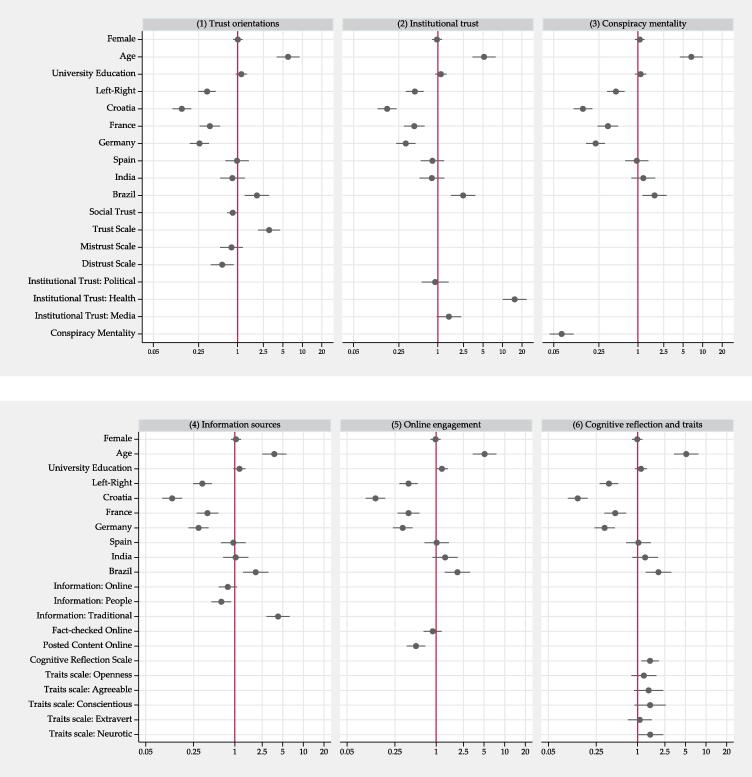
Multivariate block models of vaccine willingness, odds ratios.

The block models are displayed in Fig. 5, with effects again expressed in terms of odds ratios. The first model includes generalized trust orientations, in both government and other people, and shows that generalized trust retains its positive effect (OR = 3.08, 95 % CI 2.08 to 4.56) on vaccine willingness and distrust its negative effect (OR = 0.58, 95 % CI 0.38 to 0.87), whereas mistrust becomes a non-significant predictor. Here, social trust becomes a weakly negative predictor of willingness, albeit not significant at the 95 % level (p = 0.08), when accounting for political trust orientations. In model 2, we include measures of institutional trust, with intriguing results. Here, trust in political institutions becomes non-significant and trust in the media only significant at the 90 % level (p = 0.08), but trust in health institutions remains a strong predictor (OR = 15.37, 95 % CI 10.06 to 23.47) – suggesting again that the latter is the most robust driver of vaccine willingness. However, these measures are moderately correlated (the pairwise Pearson’s *r* coefficients are between 0.45 and 0.5) and when the other institutional trust measures are included without the others but with controls, both are significant predictors, so this should not be interpreted to mean that trust in political or media institutions does not matter for vaccine willingness. In other words, people’s general trust orientations towards government and their trust in political institutions matter for vaccine acceptance, but when accounting for their trust in different specific state institutions, it turns out that health institutions are the most closely relevant institution.

Finally, the third model shows that conspiracy mentality is an even stronger negative predictor (OR = 0.07, 95 % CI 0.04 to 0.10) when including demographic controls. Because political trust and conspiracy mentality are closely related attitudes [10],^8^ we explore whether trust is a confounder for the effects of conspiracy mentality or vice versa, by presenting the results of models including all of these variables together in Online Appendix Fig. A2: these show that the effects of conspiracy mentality survive controlling for all of the trust measures, whereas the only trust measure that still has a significant effect is that of trust in health institutions, suggesting that both shape vaccine willingness above and beyond political trust.^9^

Returning to other factors, we find that the effects of most personality traits lose their significance in the block model (neuroticism is significant at the 90 % level, p = 0.06) but here, cognitive reflection shows a positive effect on vaccine willingness (p < 0.01) and information engagement still has significant effects. In particular, the frequency of consuming traditional media (newspapers, television, radio) remains a strong predictor of vaccine willingness (OR = 4.27, 95 % CI 2.89 to 6.32), while getting information from ‘people’ and online has negative effects, although relatively weak ones (and non-significant for the former). We find that more ‘active’ internet users – those who post political content – are more vaccine hesitant (OR = 0.51, 95 % CI 0.37 to 0.69), although the effect of fact-checking becomes non-significant. We also find that moving from left to right on a left/right scale is associated with lower levels of vaccine willingness in all models. The effect of university education becomes non-significant in most models, but age retains a strong positive effect on willingness (while the effects of gender are still non-significant). The latter is perhaps unsurprising given the sharp age gradient of the severity of COVID-19.

Finally, we explore whether the determinants of vaccine hesitancy (or willingness) differ importantly between the countries in our dataset by conducting the bivariate and block models separately for each country. Coefficient plots from these models can be found in the Online Appendix, Figs. A3-A23. Most of the central findings remain the same across countries, but generalized trust orientations have by some margin the strongest relationship with vaccine hesitancy in France and Germany, whereas in Brazil political trust *negatively* predicts vaccine willingness. This finding for Brazil is consistent with other studies suggesting that partisan cues can reverse expected relationships between political trust and COVID-related attitudes and behaviours (e.g., [12]). President Bolsonaro was notoriously vaccine- and COVID-sceptic, so those respondents expressing trust in his government were more likely to also hold anti-vax attitudes. Generally, most variables have weaker and often non-significant effects in India and particularly Argentina than in the other countries, but conversely, personality traits interestingly appear to play a bigger role in those countries than in the others.

## Discussion

Our analysis here has served to demonstrate the critical link between political trust and vaccine hesitancy across the globe, demonstrating how trust in government is predictive of willingness to get vaccinated against COVID-19 for much of the world’s population. Generalized trust orientations towards government and trust in political institutions are consistent predictors of vaccine hesitancy, although when including multiple interrelated political trust measures in the same models, trust in health institutions and conspiracy mentality stand out as the most robust predictors. The design of this study has offered unprecedented global scope in its consideration of the degree to which trust in government predicts vaccine hesitancy for COVID-19 (113 countries) and systematic comparison of alternative measures of different types of trust as well as other beliefs, opinions and traits, again in a substantial cross-national design (7 countries). This allows us to conclude with increased confidence that trust is an essential resource for societies in their fight against COVID-19, and that this is true both relative to other attitudes and controlling for the demographics of individuals. Our findings confirm that willingness to be vaccinated against COVID-19 is positively linked to trust in government, and negatively linked to distrust of government. Prior cross-national studies have reported mixed results in terms of the effects of *political* trust on vaccine hesitancy, but our findings suggest that this is likely because those that report non-significant effects of political trust do so via regression models which simultaneously include various closely related measure of trust [[17], [24]]. In terms of other factors, we find that consumers of traditional media (i.e. television, newspapers) are more willing to get vaccinated, whereas the politically online (i.e. people who post on social media) are less willing. There is little evidence to support the claim that personality traits are drivers of vaccine hesitancy, even in models that exclude strong predictors such as conspiracy beliefs and political trust. We find that cognitive reflection is associated with greater vaccine willingness, but this is a small effect relative to other attitudinal and demographic predictors. Of the 29 predictors included in our regression models, political trust orientations and conspiracy mentality emerge as the strongest predictors of vaccine willingness, along with respondents’ age.

We have here built on a still growing literature examining the link between trust and vaccine hesitancy in the context of COVID-19. Our study draws upon high quality survey data from the Wellcome Global Monitor, enabling us to cover a substantially greater number of countries than most empirical studies conducted during the pandemic, and through our own cross-national survey programme, allowing us to disentangle the roles of different types of trust and to directly compare their effects with other commonly cited factors. While the COVID-19 pandemic has eased in severity by now, these results point to the importance of rebuilding political trust for the potential success of future mass vaccination programmes.

## Ethical review

This study was approved by the Faculty of Social Sciences Research Ethics Committee at the University of Southampton (Ethics/ERGO Number: 61735).

## Declaration of Competing Interest

The authors declare the following financial interests/personal relationships which may be considered as potential competing interests: Will Jennings reports financial support was provided by the UK Economic and Social Research Council (ESRC). Will Jennings reports financial support was provided by Research England QR Global Challenges Research Fund (GCRF).

## Data Availability

Data will be made available on request.

## Footnotes

1 The full wording for these questions is included in Online Appendix A.

2 Those who give ‘prefer not to say’ responses at Q13, or respond ‘don’t know’ at Q14, are dropped from the analysis.

3 In Brazil, this item specifically referred to the publicly funded healthcare system, “Sistema Único de Saúde (SUS)”.

4 Readers should bear in mind the timing of data collection (October 2020 through January 2021) and that the level of trust in each country may have increased or decreased since (for instance in Myanmar – ‘MM’ in Fig. 1 – where trust may have fallen significantly since the 2021 coup d’etat).

5 The response options were binary: “Yes, would agree” and “No, would not agree”. Those who responded “don‘t know” or refused to respond are coded as missing for the purposes of this analysis.

6 The other response options were “not much” and “not at all”. Don’t knows and refusals are once again treated as missing.

7 Here, we include the full categorical variable with dummies for each category and “not at all” as the baseline.

8 The correlation between the conspiracy mentality scale and the generalized trust scales is between 0.22 and 0.28 Pearson’s *r* in the pooled dataset, with stronger correlations in France, Germany, and Spain.

9 Generalized trust in government is still a significant predictor when including those and conspiracy mentality together in a model, but its effects become insignificant when controlling for trust in institutions as well.
