# Supplementary material for: Trust and vaccine hesitancy during the COVID-19 pandemic: A cross-national analysis
Source: Vaccine X. 2023 Apr 6;14:100299. doi: 10.1016/j.jvacx.2023.100299 (PMC10079319; doi:10.1016/j.jvacx.2023.100299)

# Online Appendix

## Appendix A: Questionnaires and Variables

### Wellcome Global Monitor

**Vaccine willingness**

“Vaccines are given to people to help prevent specific diseases. If a vaccine to prevent coronavirus was available right now at no cost, would you agree to be vaccinated?

Yes, would agree / No, would not agree / Don’t know / Refused

**Trust in government**

“How much do you trust each of the following? Do you trust them a lot, some, not much, or not at all? If you don’t know, please just say so. How about the national government in this country?

A lot / Some / Not much / Not at all/ Don’t know / Refused

### Original Survey Data

**Vaccination**

[Q13] Which of these applies to you?

<1> I have received one or two doses of a COVID-19 vaccine

<2> I have been offered a vaccine against COVID-19, but declined the offer to be vaccinated

<3> I have not yet been offered a vaccine against COVID-19

<4> Prefer not to say

[Q14] To what extent, if at all, do you agree, or disagree, with the following statements?

-[Q14_1 if Q13==3] If I was offered a vaccine for COVID-19, I would get it

<1> Strongly agree

<2> Tend to agree

<3> Neither agree nor disagree

<4> Tend to disagree

<5> Strongly disagree

<6 fixed> Don’t know

Other questions in this module:

-[Q14_1 if Q13==3] If I was offered a vaccine for COVID-19, I would get it

-[Q14_2] If my child/children were offered a vaccine for COVID-19, I would have them get it (parents only)

-[Q14_3] Data about the effectiveness of vaccines is often made-up

-[Q14_4] Vaccines are not harmful

-[Q14_5] Hot temperatures kill the COVID-19 virus

-[Q14_6] The coronavirus is no worse than the seasonal flu

**Social trust**

[Q1] {single order=randomize} Generally speaking, would you say that most people can be trusted or that you need to be very careful in dealing with people?

<1> Most people can be trusted

<2> You need to be very careful

<3 fixed> Don’t know

**Trust in health institutions**

[Q2] {grid roworder=randomize} Here is a list of organizations. How much confidence do you have in each of the following: is it a great deal of confidence, quite a lot of confidence, not very much confidence or none at all?

-[Q2_13] The World Health Organisation

-[Q2_15] The health service in your country [Brazil: O Serviço Único de Saúde (SUS)]

<1> A great deal

<2> Quite a lot

<3> Not very much

<4> None at all

<5 fixed> Don’t know

**Trust in media**

[Q19] {grid roworder=randomize} Thinking about the traditional media (i.e. newspapers and television news, including their websites), how much if at all do you agree or disagree with the following statements?

-[Q19_1] I tend to trust information in the traditional media.

<1> Strongly agree

<2> Tend to agree

<3> Neither agree nor disagree

<4> Tend to disagree

<5> Strongly disagree

<6 fixed> Don’t know

**Trust, mistrust and distrust in government**

[Q4] {grid roworder=randomize} To what extent do you agree, or disagree, with the following statements?

-[Q4_1] The government is honest and truthful [TRUST]

-[Q4_2] In general, the government usually does the right thing [TRUST]

-[Q4_3] The government usually has good intentions [TRUST]

-[Q4_4] I usually trust the government instinctively [MISTRUST – REVERSED]

-[Q4_5] I am usually cautious about trusting the government [MISTRUST]

-[Q4_6] I am unsure whether to believe the government [MISTRUST]

-[Q4_7] The government acts unfairly towards people like me [DISTRUST]

-[Q4_8] The government usually ignores my community [DISTRUST]

-[Q4_9] The government doesn’t respect people like me [DISTRUST]

-[Q4_10] The government cares less about people in my area than people in other parts of the country [DISTRUST]

<1> Strongly agree

<2> Tend to agree

<3> Neither agree nor disagree

<4> Tend to disagree

<5> Strongly disagree

<6> Don’t know

**Conspiracy mentality**

[Q15] {grid roworder=randomize} What do you think the likelihood is of each of the following being true or not true? Please use a scale of 0 to 10, where 0 is certainly not true, and 10 is certainly true.

-[Q15_1] …many very important things happen in the world, which the public is never informed about

-[Q15_2] …politicians usually do not tell us the true motives for their decisions

-[Q15_3] …government agencies closely monitor all citizens

-[Q15_4] …events which superficially seem to lack a connection are often the result of secret activities

-[Q15_5] …there are secret organisations that greatly influence political decisions

-[Q15_7] …much of what happens in the world today is decided by a small and secretive group of individuals

<1> 0 - Certainly not true

<2> 1

<3> 2

<4> 3

<5> 4

<6> 5 - About 50/50

<7> 6

<8> 7

<9> 8

<10> 9

<11> 10 - Certainly true

<12> Don’t know

**Information sources**

[Q17] During the last month, on average how much time (if any) have you spent following news about politics or current affairs from each of these sources?

-[Q17_1] Television [INFO: TRADITIONAL]

-[Q17_2] Newspaper (including online) [INFO: TRADITIONAL]

-[Q17_3] Radio [INFO: TRADITIONAL]

-[Q17_4] Internet (not including online newspapers) [INFO: ONLINE]

-[Q17_5] Talking to other people [INFO: PEOPLE]

<1> None, no time at all

<2> At least once in the past month

<3> Once a week

<4> Several times a week

<5> Once a day

<6> Several times a day

<7 fixed> Don’t know

**Ideology**

[Q30x] In political matters, people talk of "the left" and "the right." How would you place your views on this scale, generally speaking?

<1> 1 - Left

<2> 2

<3> 3

<4> 4

<5> 5

<6> 6

<7> 7

<8> 8

<9> 9

<10> Right - 10

**Cognitive reflection**

[cognitive1x] A bat and a ball cost $1.10 in total. The bat costs $1.00 more than the ball. How much does the ball cost? Please enter the number of cents below. <<br/>> *Each dollar ($) is worth 100 cents.*

<1> [NUMBER – range between 0 and 10,000]

<2> Don’t Know

[cognitive2x] If it takes five machines 5 min to make five widgets, how long would it take 100 machines to make 100 widgets? Please enter the number of minutes below.

<1> [NUMBER – range between 0 and 10,000]

<2> Don’t Know

[cognitive3x] In a lake, there is a patch of lily pads. Every day, the patch doubles in size. If it takes 48 days for the patch to cover the entire lake, how long would it take for the patch to cover half of the lake? Please enter the number of days below.

<1> [NUMBER – range between 0 and 10,000]

<2> Don’t Know

**Personality traits**

[Q26.] I see myself as someone who. . .

1. ...is original, comes up with new ideas

2. ...values artistic, aesthetic experiences

3. ...has an active imagination

4. ...is sometimes rude to others

5. ...has a forgiving nature

6. ...is considerate and kind to almost everyone

7. ...does a thorough job

8. ...tends to be lazy

9. ...does things efficiently

10. ...is talkative

11. ...is outgoing, sociable

12. ...is reserved

13. ...worries a lot

14. ...gets nervous easily

15. ...remains calm in tense situations

<1> Strongly agree

<2> Tend to agree

<3> Neither agree nor disagree

<4> Tend to disagree

<5> Strongly disagree

<6> Don’t know

**Demographic and political controls**

Specific questions and categories vary between countries, based on each survey company’s pre-existing panel data

1. Age in years
2. Sex (male=0, female=1)
3. University education (coded as 1 if respondent had completed some university education)

**Table M1.** Detailed coding of variables in the analysis

| **Measure(s)** | **Coding** | **Question(s)** |
| --- | --- | --- |
| Vaccine willingness | 0 = unwilling  1 = willing  If Q13 = 1, code 1  If Q13 = 2, code 0  If Q13 = 3:  Code 1 if Q14_1 = 1-2  Code 0 if Q14_1 = 3-5  Code missing if Q14_1 = 6  If Q13 = 4: code missing. | Q13, Q14 (1) |
| Social trust | 0 = You need to be very careful / Don’t know  1 = Most people can be trusted | Q1 |
| Trust: government | 1 = Strongly disagree  2 = Tend to disagree  3 = Neither agree nor disagree  4 = Tend to agree  5 = Strongly agree  Alpha index of mean agreement, rescaled: 0 to 1. | Q4 (1-3) |
| Mistrust: government | 1 = Strongly disagree  2 = Tend to disagree  3 = Neither agree nor disagree  4 = Tend to agree  5 = Strongly agree  Alpha index of mean agreement, rescaled: 0 to 1. | Q4 (4-6) |
| Distrust: government | 1 = Strongly disagree  2 = Tend to disagree  3 = Neither agree nor disagree  4 = Tend to agree  5 = Strongly agree  Alpha index of mean agreement, rescaled: 0 to 1. | Q4 (7-10) |
| Trust: health organisations  (Country’s health service, WHO) | 1 = None at all  2 = Not very much  3 = Quite a lot  4 = A great deal  Alpha index of mean agreement, rescaled: 0 to 1. | Q2 (13, 15) |
| Trust: media | 1 = Strongly disagree  2 = Tend to disagree  3 = Neither agree nor disagree  4 = Tend to agree  5 = Strongly agree  Rescaled: 0 to 1. | Q19 (1) |
| Conspiracy mentality | 0 = Certainly not true  1  2  3  4  5 = About 50/50  6  7  8  9  10 = Certainly true  Alpha index of mean agreement, rescaled: 0 to 1. | Q15 (1-5, 7) |
| Information sources: online | 1 = None, no time at all  2 = At least once in the past month  3 = Once a week  4 = Several times a week  5 = Once a day  6 = Several times a day  Rescaled: 0 to 1. | Q17 (4) |
| Information sources: people | 1 = None, no time at all  2 = At least once in the past month  3 = Once a week  4 = Several times a week  5 = Once a day  6 = Several times a day  Rescaled: 0 to 1. | Q17 (5) |
| Information sources: traditional | 1 = None, no time at all  2 = At least once in the past month  3 = Once a week  4 = Several times a week  5 = Once a day  6 = Several times a day  Rescaled: 0 to 1. | Q7 (1-3) |
| Posted political content online | 0 = No  1 = Yes (posted information about politics or current affairs on social media in the past month) | Q18 (3) |
| Fact-checked an article online | 0 = No  1 = Yes (‘fact-checked’ an article about politics or current affairs in the past month) | Q18 (2) |
| Ideology | 0-10 scale (0 = left, 10=right).  Rescaled: 0 to 1. | Q30x |
| Cognitive reflection | If cognitive1x = 5, score 1, else: 0.  If cognitive2x = 5, score 1, else: 0  If cognitive3x = 47, score 1, else: 0  Summed and rescaled: 0 to 1. | cognitive1x, cognitive2x, cognitive3x |
| Personality: openness | <1> Strongly agree  <2> Tend to agree  <3> Neither agree nor disagree  <4> Tend to disagree  <5> Strongly disagree  <6> Don’t know | Q32 (1-3) |
| Personality: conscientiousness | <1> Strongly agree  <2> Tend to agree  <3> Neither agree nor disagree  <4> Tend to disagree  <5> Strongly disagree  <6> Don’t know | Q32 (4-6) |
| Personality: extraversion | <1> Strongly agree  <2> Tend to agree  <3> Neither agree nor disagree  <4> Tend to disagree  <5> Strongly disagree  <6> Don’t know | Q32 (7-9) |
| Personality: agreeableness | <1> Strongly agree  <2> Tend to agree  <3> Neither agree nor disagree  <4> Tend to disagree  <5> Strongly disagree  <6> Don’t know | Q32 (10-12) |
| Personality: neuroticism | <1> Strongly agree  <2> Tend to agree  <3> Neither agree nor disagree  <4> Tend to disagree  <5> Strongly disagree  <6> Don’t know | Q32 (13-15) |
| Female | 0 = No  1 = Yes |  |
| Age | Continuous variable, rescaled: 0 to 1. |  |
| Graduate | 0 = No degree  1 = Degree or higher qualification |  |

## Appendix B: Tables and Figures

**Table A1.** Multi-level regression model of vaccine intention in 91 countries. Source: 2020 Global Wellcome Monitor data.

|  | (1) |
| --- | --- |
|  | Vaccine Intention |
|  |  |
| Base (Not at all) | . |
|  |  |
| Not much | 0.071^***^ (0.005) |
|  |  |
| Some | 0.124^***^ (0.004) |
|  |  |
| A lot | 0.174^***^ (0.005) |
|  |  |
| Base (15-29) | . |
|  |  |
| 30-49 | -0.007^+^ (0.004) |
|  |  |
| 50-64 | 0.041^***^ (0.005) |
|  |  |
| 65+ | 0.114^***^ (0.006) |
|  |  |
| Female | -0.048^***^ (0.003) |
|  |  |
| University Education | 0.018^***^ (0.003) |
|  |  |
| Employed | -0.003 (0.003) |
|  |  |
| Income Quintiles | 0.003^**^ (0.001) |
|  |  |
| Human Development Index | 0.050^+^ (0.026) |
|  |  |
| Constant | 0.488^***^ (0.026) |
| Level 2: Country |  |
| Constant | 0.022^***^ (0.003) |
| Level 3:  Global region |  |
| Constant |  |
| Residual variance | 0.203^***^ (0.001) |
| Observations | 94620 |

Standard errors in parentheses

^+^ *p* < 0.10, ^*^ *p* < 0.05, ^**^ *p* < 0.01, ^***^ *p* < 0.001

**Table A2.** Multivariate (‘block’) regression models of vaccine acceptance in seven countries, part 1.

|  | (1) | (2) | (3) |
| --- | --- | --- | --- |
|  | Trust orientations | Institutional trust | Conspiracy mentality |
|  |  |  |  |
| Female | 0.008 (0.085) | -0.028 (0.087) | 0.066 (0.086) |
|  |  |  |  |
| Age | 1.795^***^ (0.206) | 1.647^***^ (0.209) | 1.900^***^ (0.208) |
|  |  |  |  |
| University Education | 0.131 (0.102) | 0.111 (0.105) | 0.096 (0.104) |
|  |  |  |  |
| Left-Right | -1.095^***^ (0.156) | -0.818^***^ (0.160) | -0.788^***^ (0.156) |
|  |  |  |  |
| Argentina | 0.000 (.) | 0.000 (.) | 0.000 (.) |
|  |  |  |  |
| Croatia | -1.988^***^ (0.169) | -1.801^***^ (0.172) | -1.958^***^ (0.169) |
|  |  |  |  |
| France | -0.987^***^ (0.186) | -0.836^***^ (0.187) | -1.067^***^ (0.185) |
|  |  |  |  |
| Germany | -1.360^***^ (0.173) | -1.138^***^ (0.174) | -1.505^***^ (0.172) |
|  |  |  |  |
| Spain | -0.022 (0.211) | -0.190 (0.213) | -0.041 (0.211) |
|  |  |  |  |
| India | -0.188 (0.224) | -0.211 (0.225) | 0.193 (0.217) |
|  |  |  |  |
| Brazil | 0.633^**^ (0.220) | 0.872^***^ (0.220) | 0.560^*^ (0.218) |
|  |  |  |  |
| Social Trust | -0.176^+^ (0.100) |  |  |
|  |  |  |  |
| Trust Scale | 1.126^***^ (0.200) |  |  |
|  |  |  |  |
| Mistrust Scale | -0.218 (0.204) |  |  |
|  |  |  |  |
| Distrust Scale | -0.551^**^ (0.209) |  |  |
|  |  |  |  |
| Institutional Trust: Political |  | -0.089 (0.244) |  |
|  |  |  |  |
| Institutional Trust: Health |  | 2.732^***^ (0.216) |  |
|  |  |  |  |
| Institutional Trust: Media |  | 0.393^+^ (0.222) |  |
|  |  |  |  |
| Conspiracy Mentality |  |  | -2.711^***^ (0.218) |
|  |  |  |  |
| Constant | 2.796^***^ (0.293) | 1.096^***^ (0.250) | 4.219^***^ (0.274) |
| Observations | 6235 | 6235 | 6235 |

Standard errors in parentheses

^+^ *p* < 0.10, ^*^ *p* < 0.05, ^**^ *p* < 0.01, ^***^ *p* < 0.001

**Table A3.** Multivariate (‘block’) regression models of vaccine acceptance in seven countries, part 2.

|  | (4) | (5) | (6) |
| --- | --- | --- | --- |
|  | Information sources | Online engagement | Cognitive reflection and traits |
|  |  |  |  |
| Female | 0.036 (0.085) | -0.022 (0.085) | -0.012 (0.087) |
|  |  |  |  |
| Age | 1.328^***^ (0.207) | 1.628^***^ (0.203) | 1.637^***^ (0.209) |
|  |  |  |  |
| University Education | 0.155 (0.102) | 0.191^+^ (0.102) | 0.116 (0.102) |
|  |  |  |  |
| Left-Right | -1.092^***^ (0.156) | -0.933^***^ (0.155) | -0.966^***^ (0.156) |
|  |  |  |  |
| Argentina | 0.000 (.) | 0.000 (.) | 0.000 (.) |
|  |  |  |  |
| Croatia | -2.106^***^ (0.170) | -2.041^***^ (0.168) | -2.012^***^ (0.170) |
|  |  |  |  |
| France | -0.921^***^ (0.185) | -0.930^***^ (0.186) | -0.751^***^ (0.186) |
|  |  |  |  |
| Germany | -1.220^***^ (0.170) | -1.126^***^ (0.168) | -1.107^***^ (0.172) |
|  |  |  |  |
| Spain | -0.050 (0.210) | 0.015 (0.209) | 0.028 (0.210) |
|  |  |  |  |
| India | 0.028 (0.216) | 0.302 (0.215) | 0.254 (0.219) |
|  |  |  |  |
| Brazil | 0.659^**^ (0.218) | 0.676^**^ (0.217) | 0.663^**^ (0.218) |
|  |  |  |  |
| Information: Online | -0.237 (0.156) |  |  |
|  |  |  |  |
| Information: People | -0.453^**^ (0.170) |  |  |
|  |  |  |  |
| Information: Traditional | 1.453^***^ (0.199) |  |  |
|  |  |  |  |
| Fact-checked Online |  | -0.117 (0.153) |  |
|  |  |  |  |
| Posted Content Online |  | -0.679^***^ (0.158) |  |
|  |  |  |  |
| Cognitive Reflection Scale |  |  | 0.419^**^ (0.149) |
|  |  |  |  |
| Traits scale: Openness |  |  | 0.210 (0.214) |
|  |  |  |  |
| Traits scale: Agreeable |  |  | 0.372 (0.249) |
|  |  |  |  |
| Traits scale: Conscientious |  |  | 0.423 (0.267) |
|  |  |  |  |
| Traits scale: Extravert |  |  | 0.074 (0.204) |
|  |  |  |  |
| Traits scale: Neurotic |  |  | 0.426^+^ (0.223) |
|  |  |  |  |
| Constant | 2.410^***^ (0.247) | 2.760^***^ (0.238) | 1.540^***^ (0.325) |
| Observations | 6235 | 6235 | 6235 |

Standard errors in parentheses

^+^ *p* < 0.10, ^*^ *p* < 0.05, ^**^ *p* < 0.01, ^***^ *p* < 0.001

**Table A4.** Full multivariate regression models of vaccine acceptance in seven countries.

|  | (1) |
| --- | --- |
|  | Full model |
|  |  |
| Social Trust | -0.335^**^ (0.107) |
|  |  |
| Trust Scale | 0.186 (0.259) |
|  |  |
| Mistrust Scale | -0.389^+^ (0.221) |
|  |  |
| Distrust Scale | -0.101 (0.224) |
|  |  |
| Institutional Trust: Political | -0.521^+^ (0.310) |
|  |  |
| Institutional Trust: Health | 2.428^***^ (0.223) |
|  |  |
| Institutional Trust: Media | 0.237 (0.230) |
|  |  |
| Conspiracy Mentality | -2.181^***^ (0.249) |
|  |  |
| Female | -0.083 (0.093) |
|  |  |
| Age | 1.498^***^ (0.230) |
|  |  |
| University Education | 0.051 (0.110) |
|  |  |
| Information: Online | -0.081 (0.171) |
|  |  |
| Information: People | -0.235 (0.182) |
|  |  |
| Information: Traditional | 1.208^***^ (0.217) |
|  |  |
| Fact-checked Online | -0.287^+^ (0.172) |
|  |  |
| Posted Content Online | -0.666^***^ (0.173) |
|  |  |
| Cognitive Reflection Scale | 0.249 (0.161) |
|  |  |
| Traits scale: Openness | 0.542^*^ (0.231) |
|  |  |
| Traits scale: Agreeable | -0.018 (0.266) |
|  |  |
| Traits scale: Conscientious | 0.670^*^ (0.289) |
|  |  |
| Traits scale: Extravert | 0.071 (0.220) |
|  |  |
| Traits scale: Neurotic | 0.767^**^ (0.238) |
|  |  |
| Left-Right | -0.586^***^ (0.164) |
|  |  |
| Argentina | Ref. |
|  |  |
| Croatia | -1.964^***^ (0.180) |
|  |  |
| France | -0.960^***^ (0.198) |
|  |  |
| Germany | -1.268^***^ (0.185) |
|  |  |
| Spain | -0.127 (0.218) |
|  |  |
| India | -0.125 (0.240) |
|  |  |
| Brazil | 0.655^**^ (0.229) |
|  |  |
| Constant | 1.849^***^ (0.399) |
| Observations | 6235 |

Standard errors in parentheses

^+^ *p* < 0.10, ^*^ *p* < 0.05, ^**^ *p* < 0.01, ^***^ *p* < 0.001

**Figure A1.** Full multivariate regression, coefficient plot using results from Table A4.


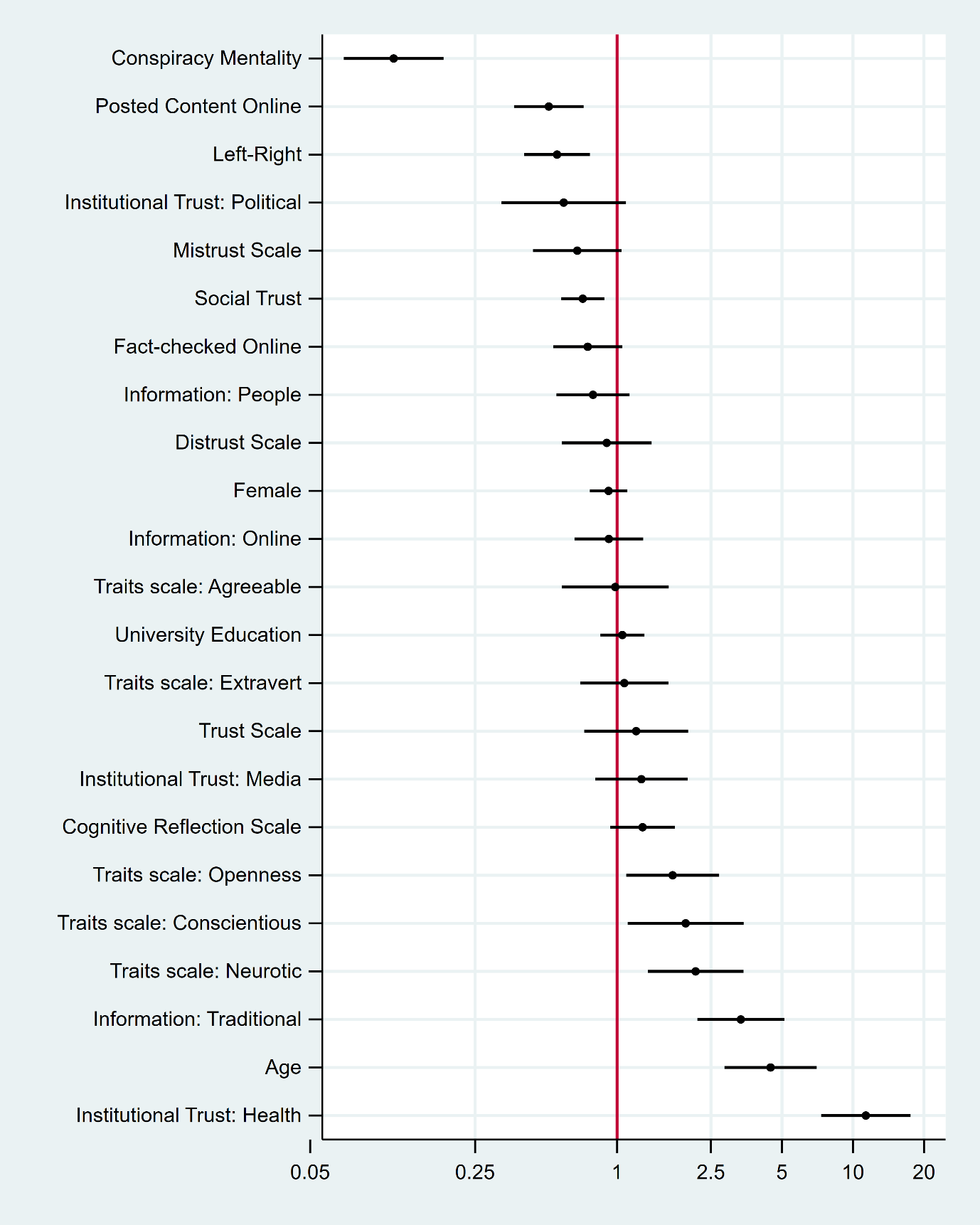


(Note: The coefficients for country dummy variables are dropped from this plot.)

**Figure A2.** Multivariate block models including conspiracy mentality and different trust measures together


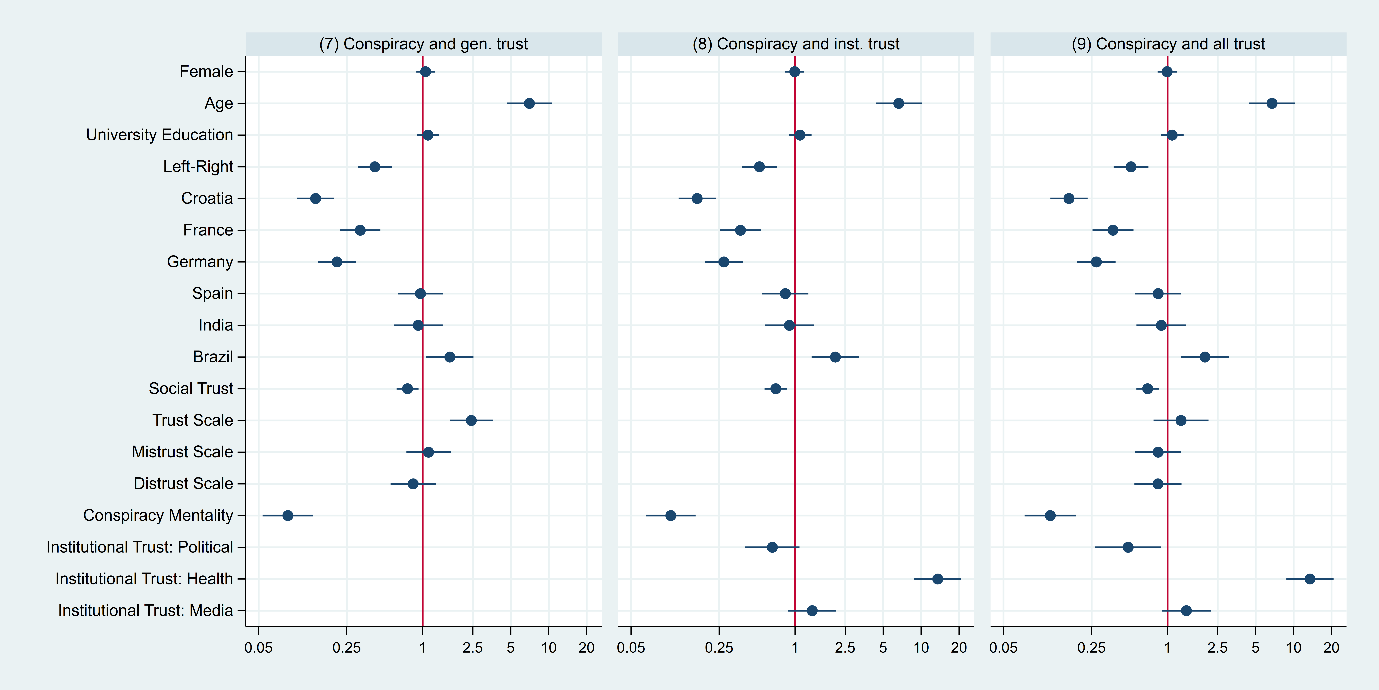


**Table A5.** Pairwise correlations (Pearson’s r) between different measures of trust and conspiracy mentality in the pooled dataset of seven countries.

|  | **Conspiracy Mentality** | **Social Trust** | **Trust Scale** | **Mistrust Scale** | **Distrust Scale** | **Institutional Trust: Political** | **Institutional Trust: Health** | **Institutional Trust: Media** |
| --- | --- | --- | --- | --- | --- | --- | --- | --- |
| **Conspiracy Mentality** | 1.00 |  |  |  |  |  |  |  |
| **Social Trust** | -0.17 | 1.00 |  |  |  |  |  |  |
| **Trust Scale** | -0.23 | 0.24 | 1.00 |  |  |  |  |  |
| **Mistrust Scale** | 0.25 | -0.14 | -0.37 | 1.00 |  |  |  |  |
| **Distrust Scale** | 0.28 | -0.15 | -0.49 | 0.55 | 1.00 |  |  |  |
| **Institutional Trust: Political** | -0.24 | 0.30 | 0.69 | -0.32 | -0.37 | 1.00 |  |  |
| **Institutional Trust: Health** | -0.19 | 0.20 | 0.33 | -0.08 | -0.15 | 0.48 | 1.00 |  |
| **Institutional Trust: Media** | -0.17 | 0.21 | 0.31 | -0.07 | -0.06 | 0.49 | 0.45 | 1.00 |

**Figures A3-A9.** Bivariate regressions of variables and vaccine willingness, by country


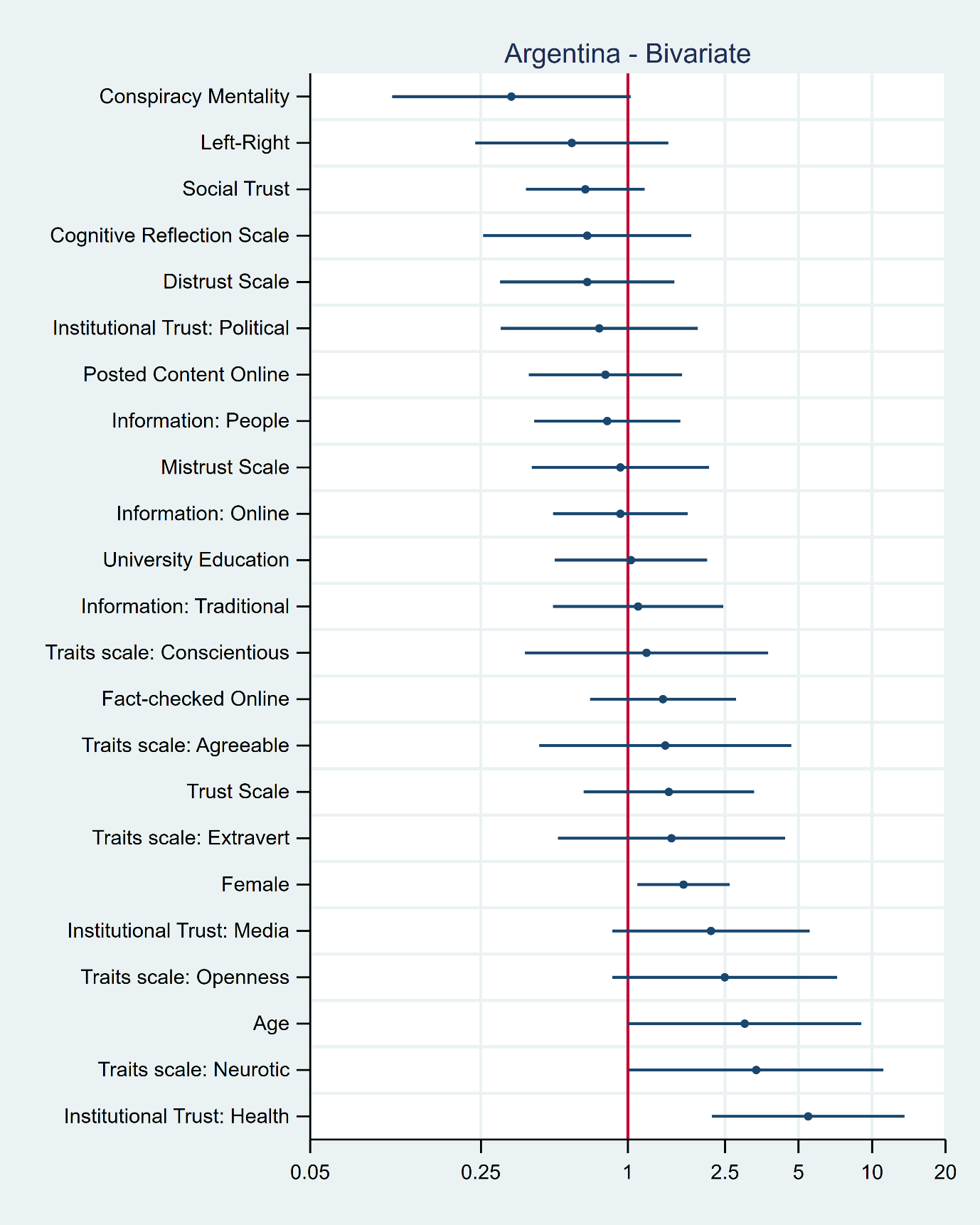


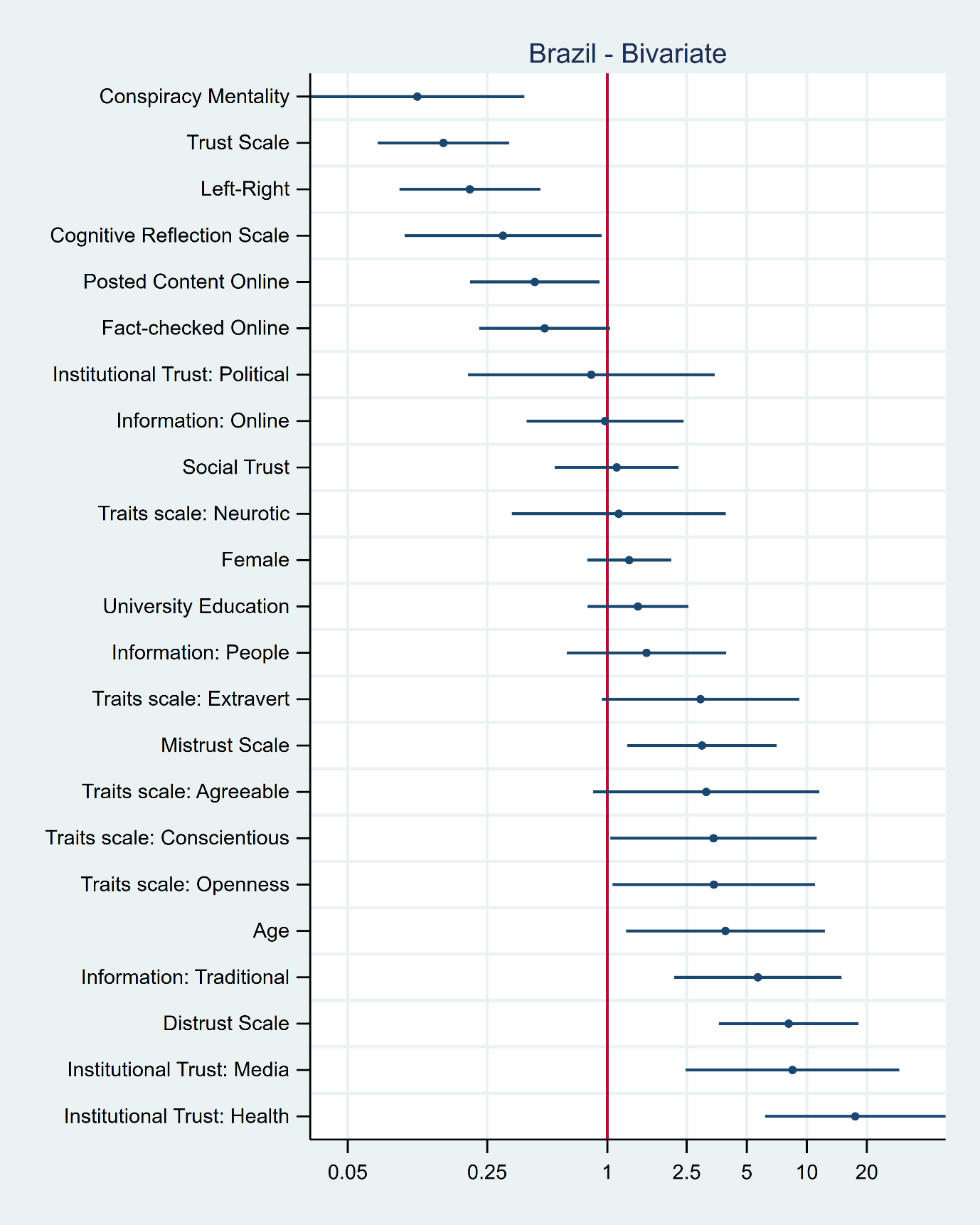


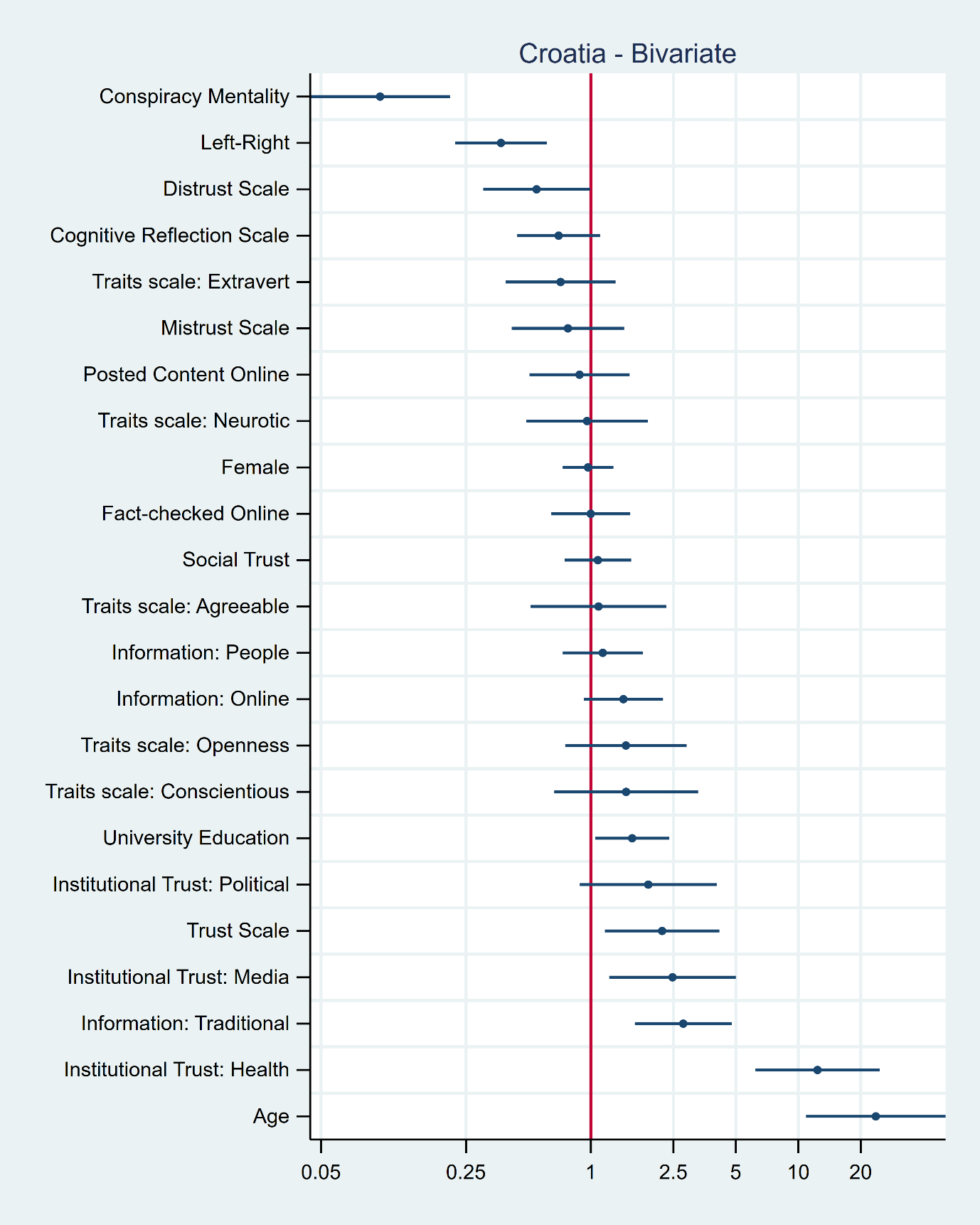


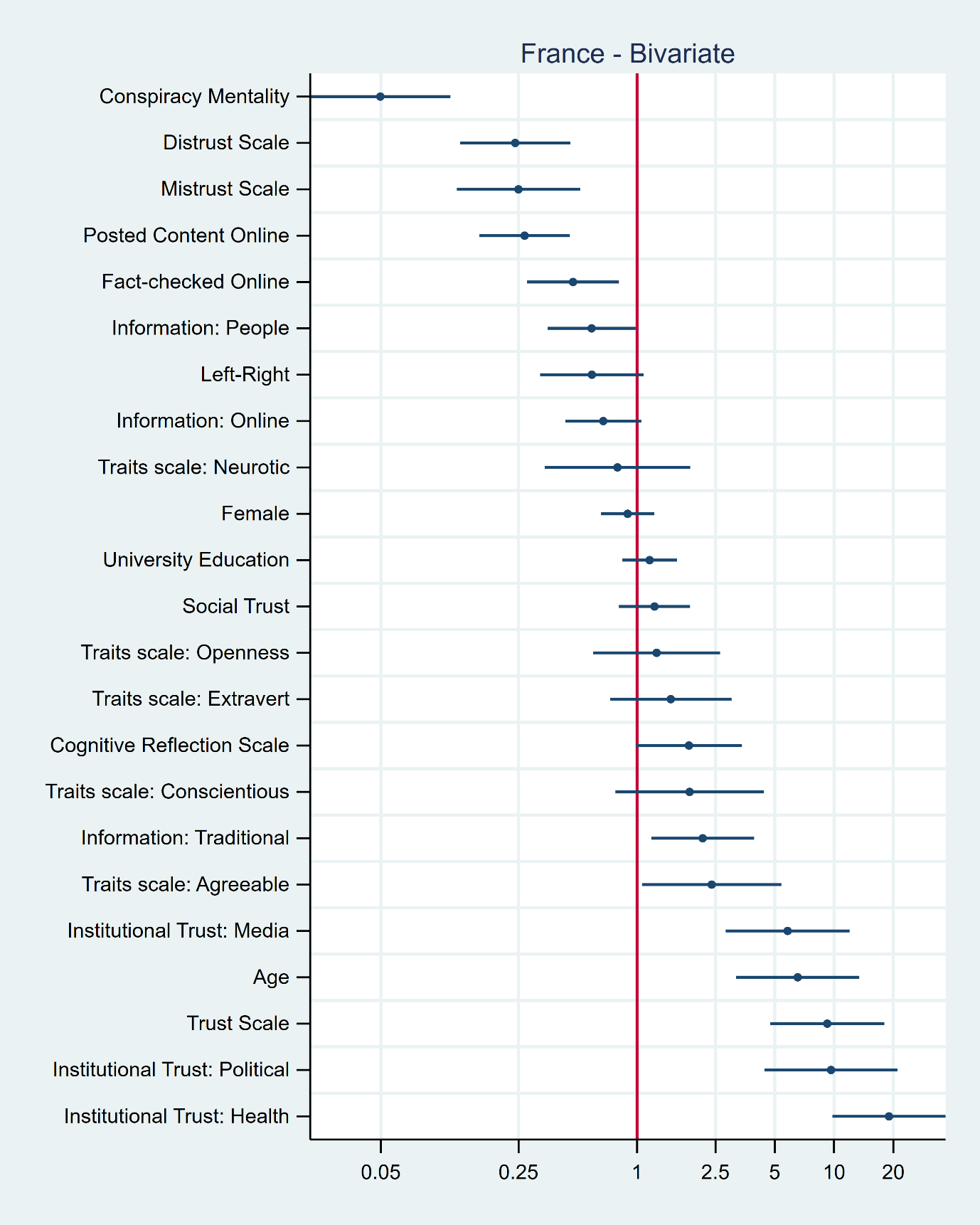


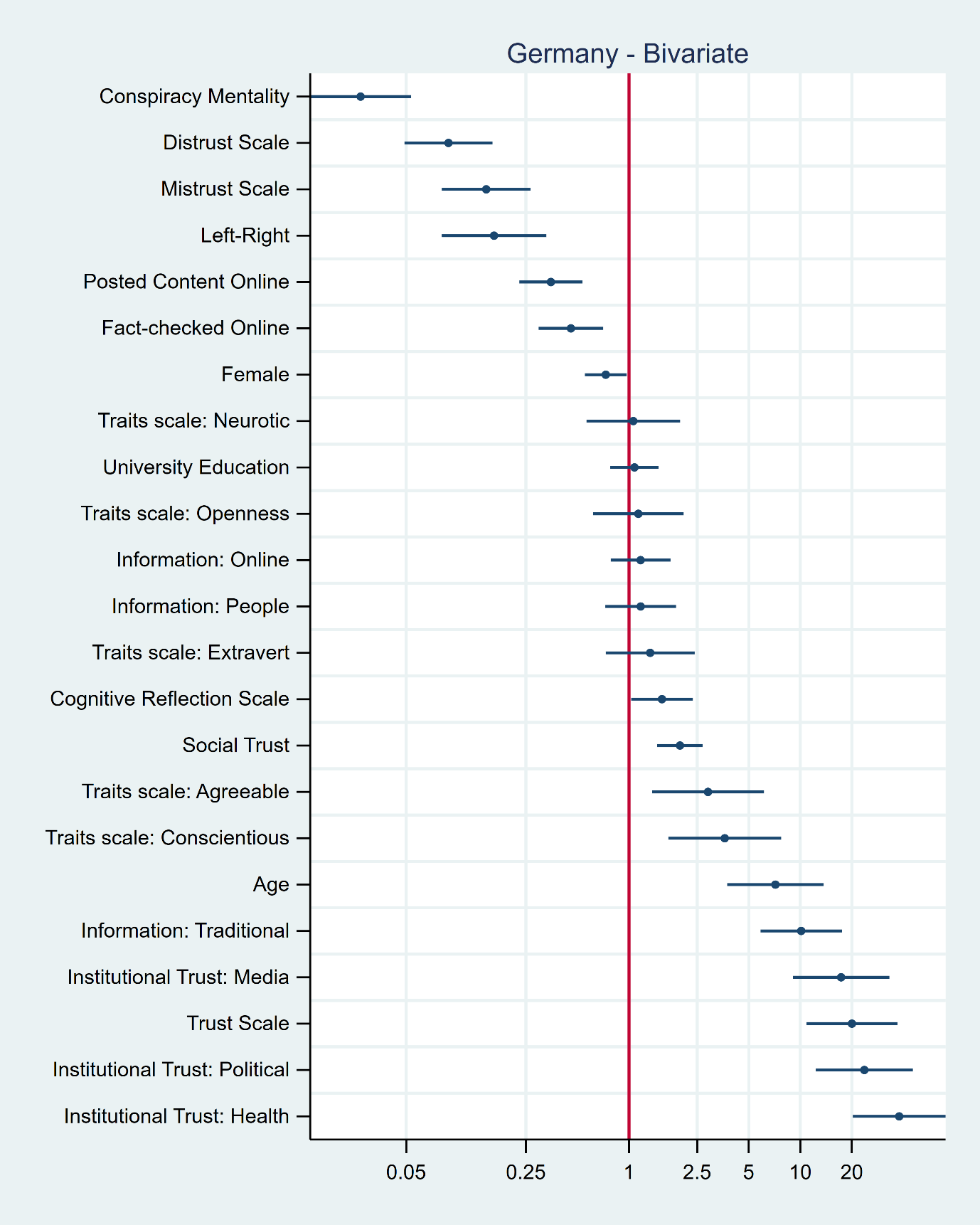


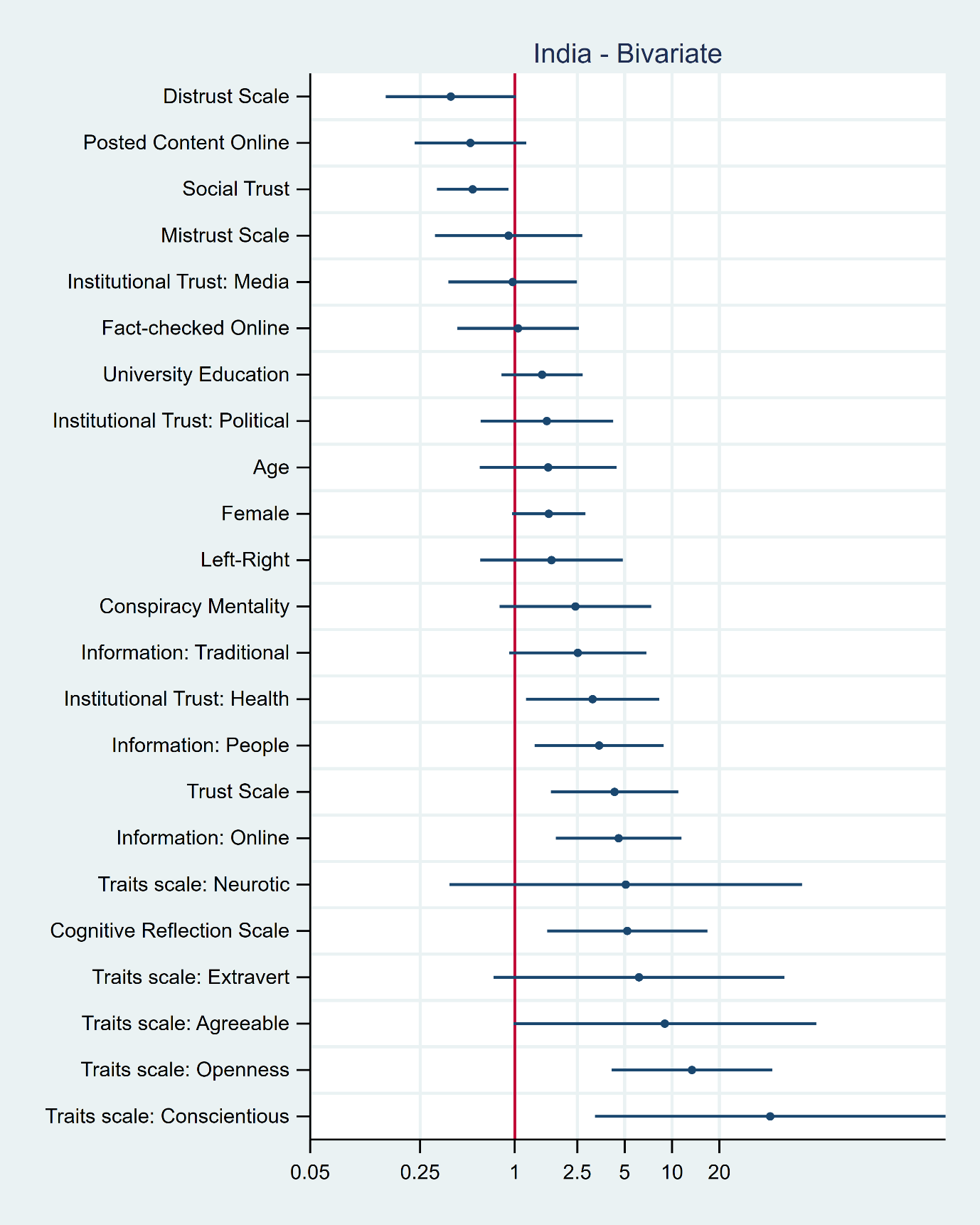


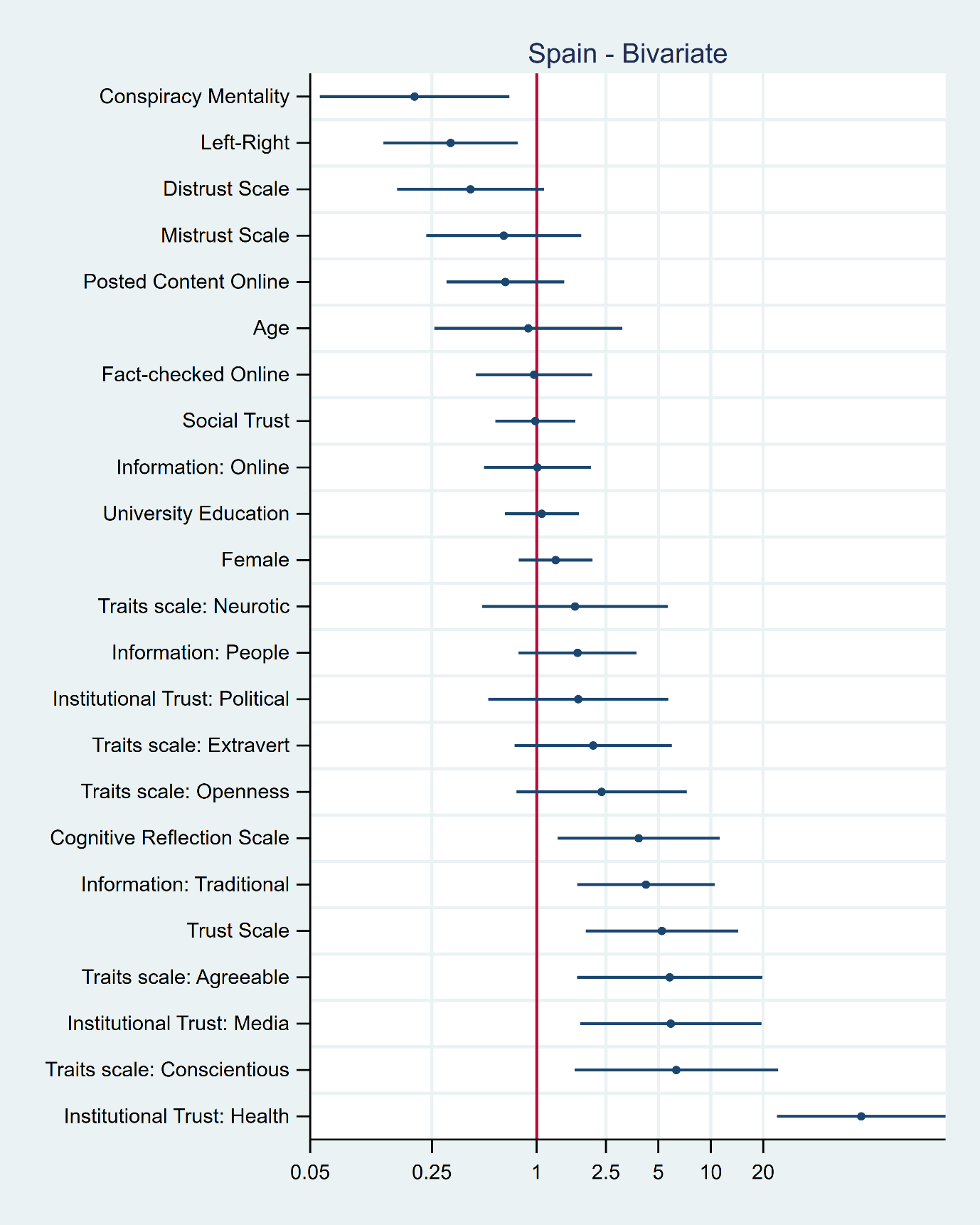


**Figures A10-A23.** Multivariate regressions of variables and vaccine willingness, by country


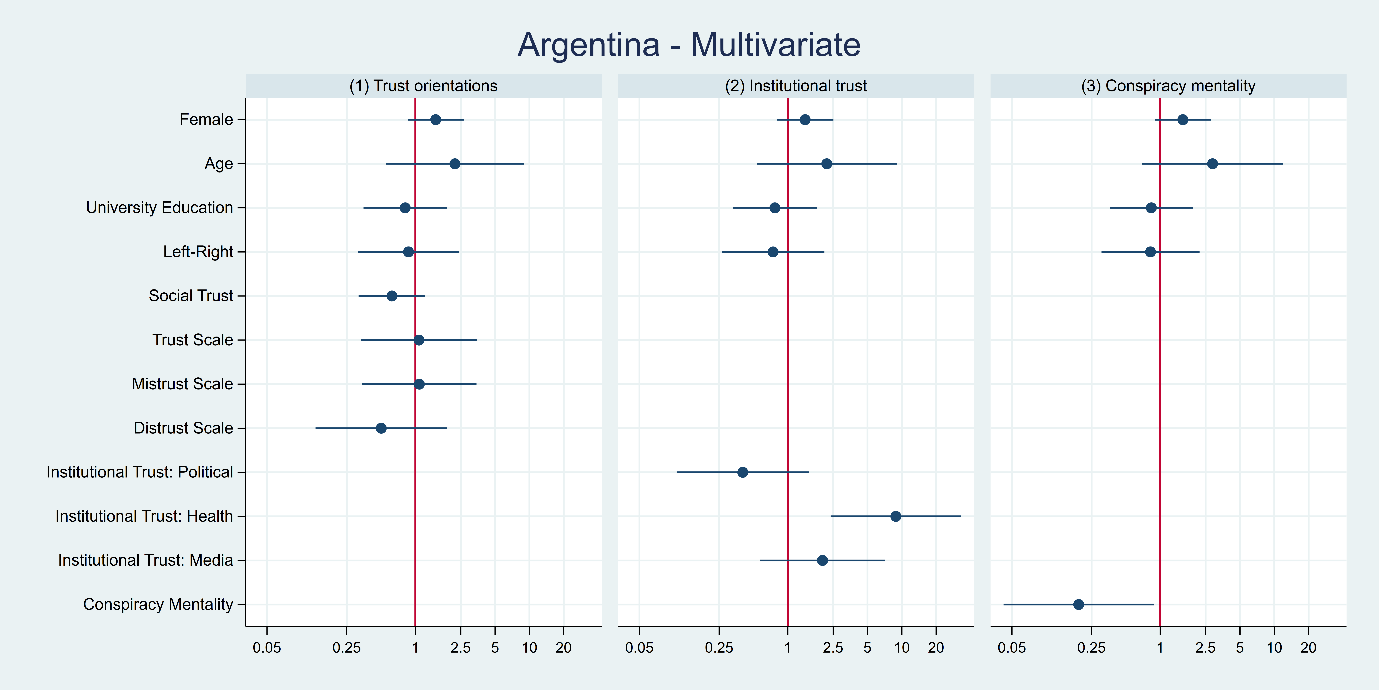


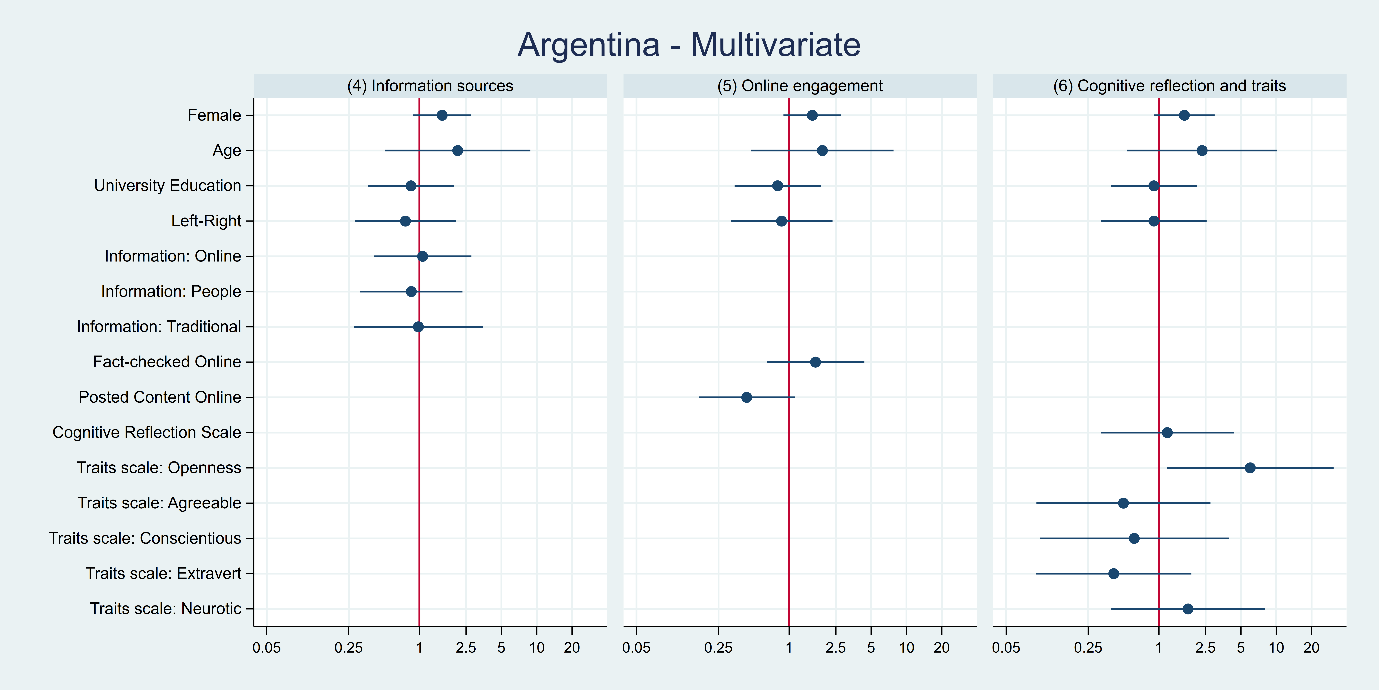


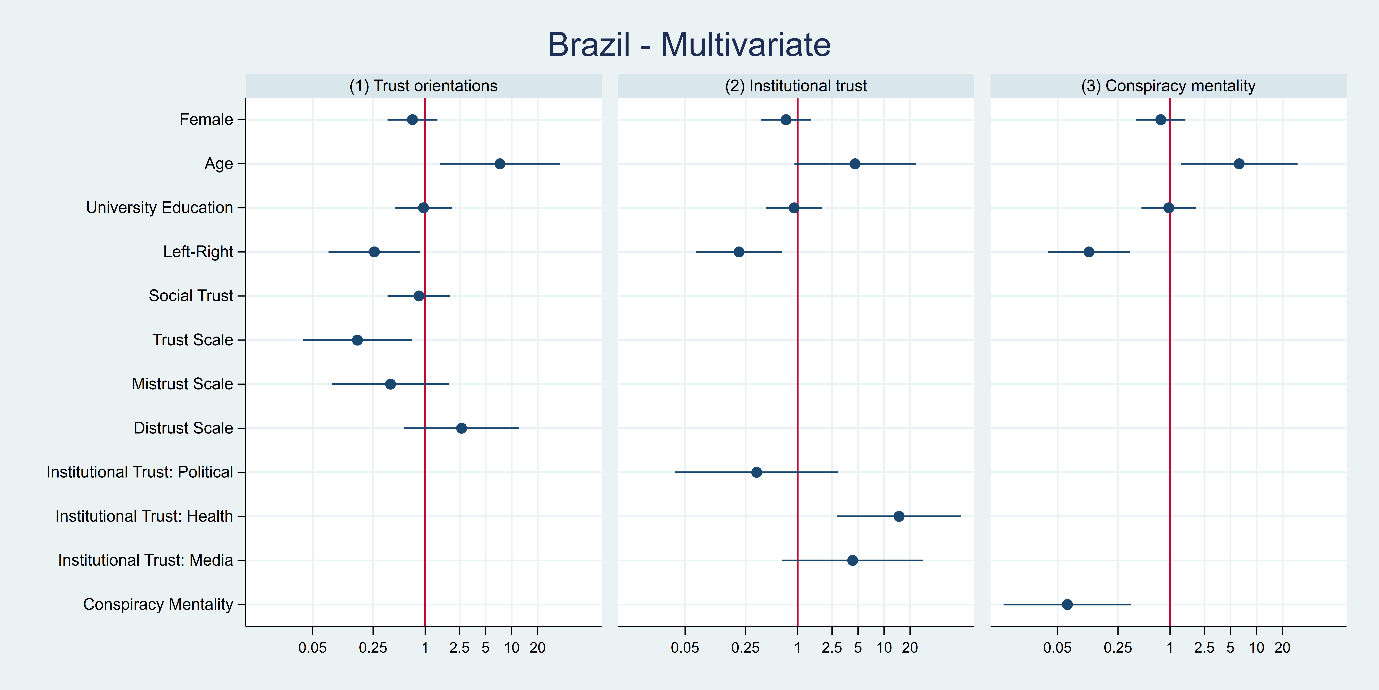


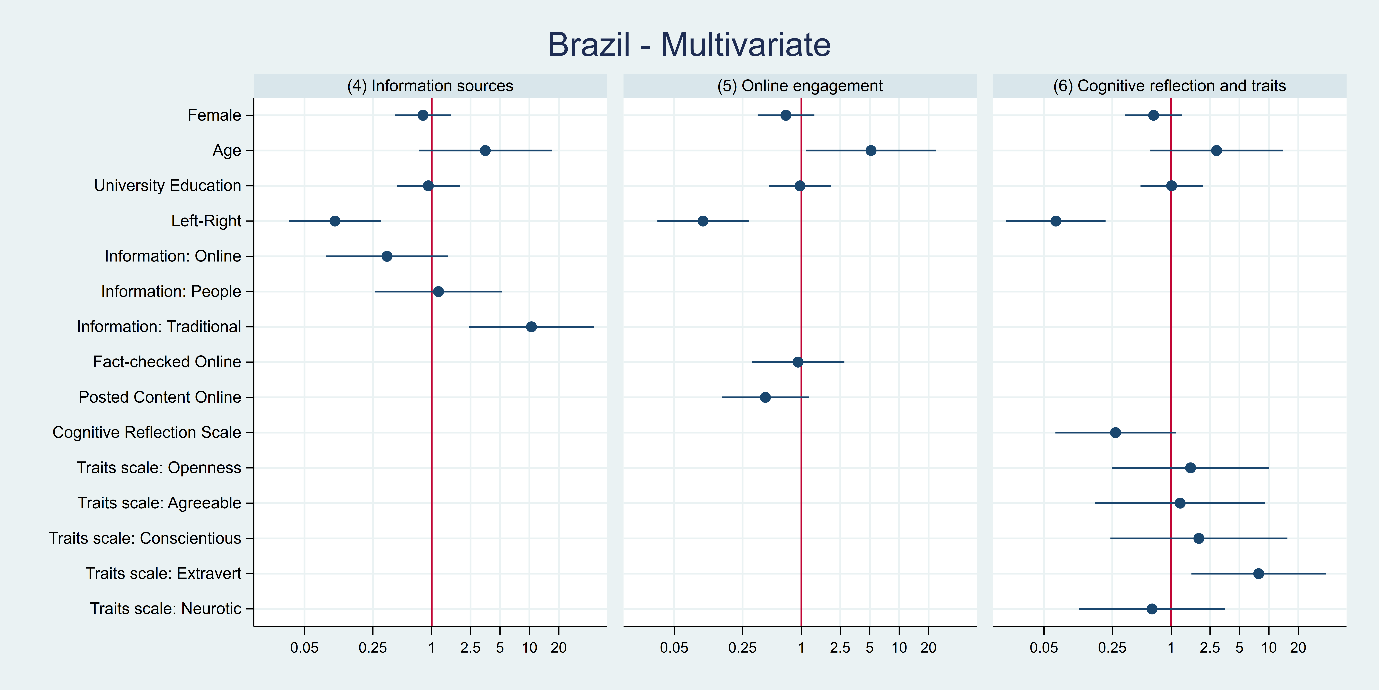


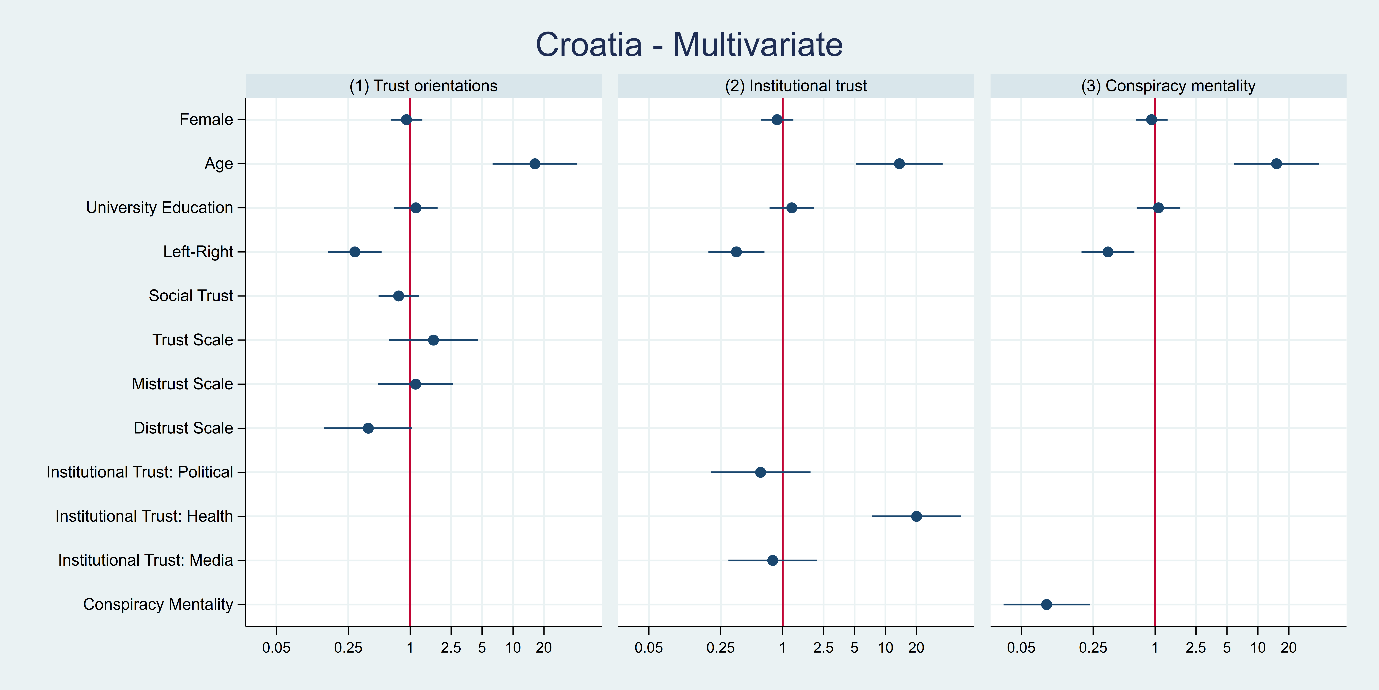


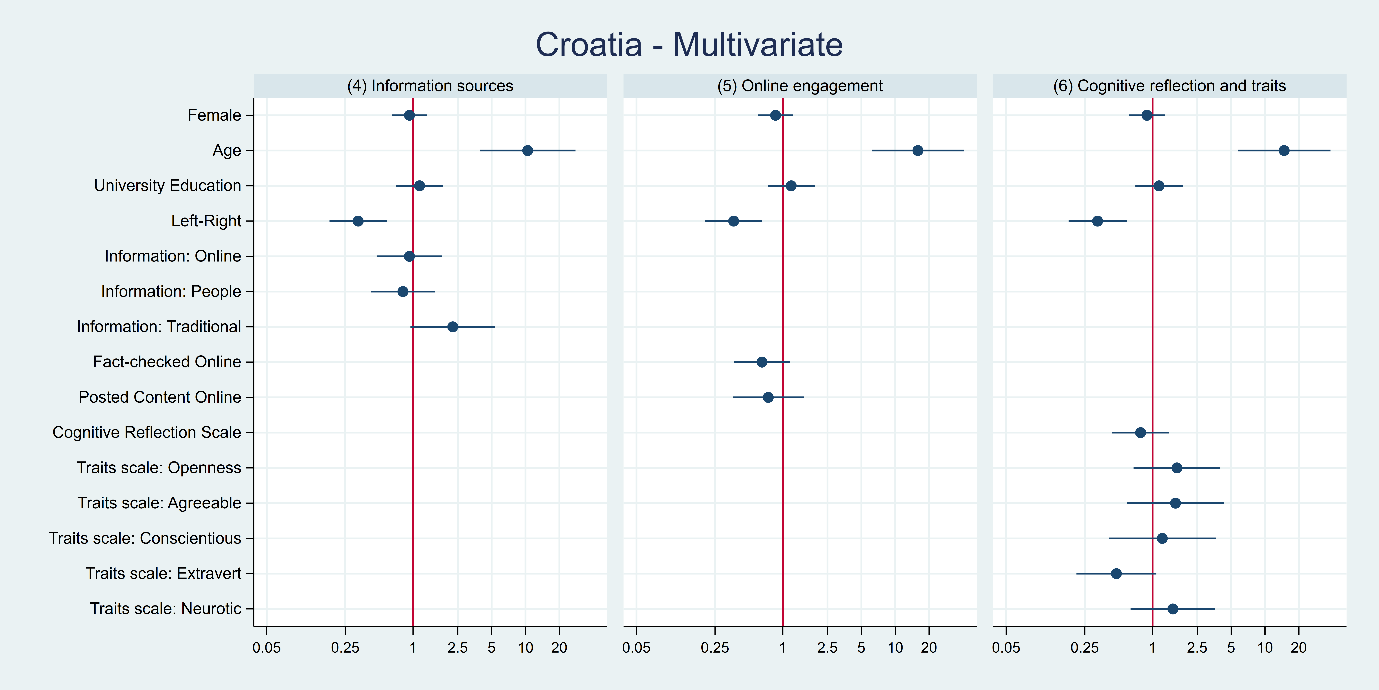


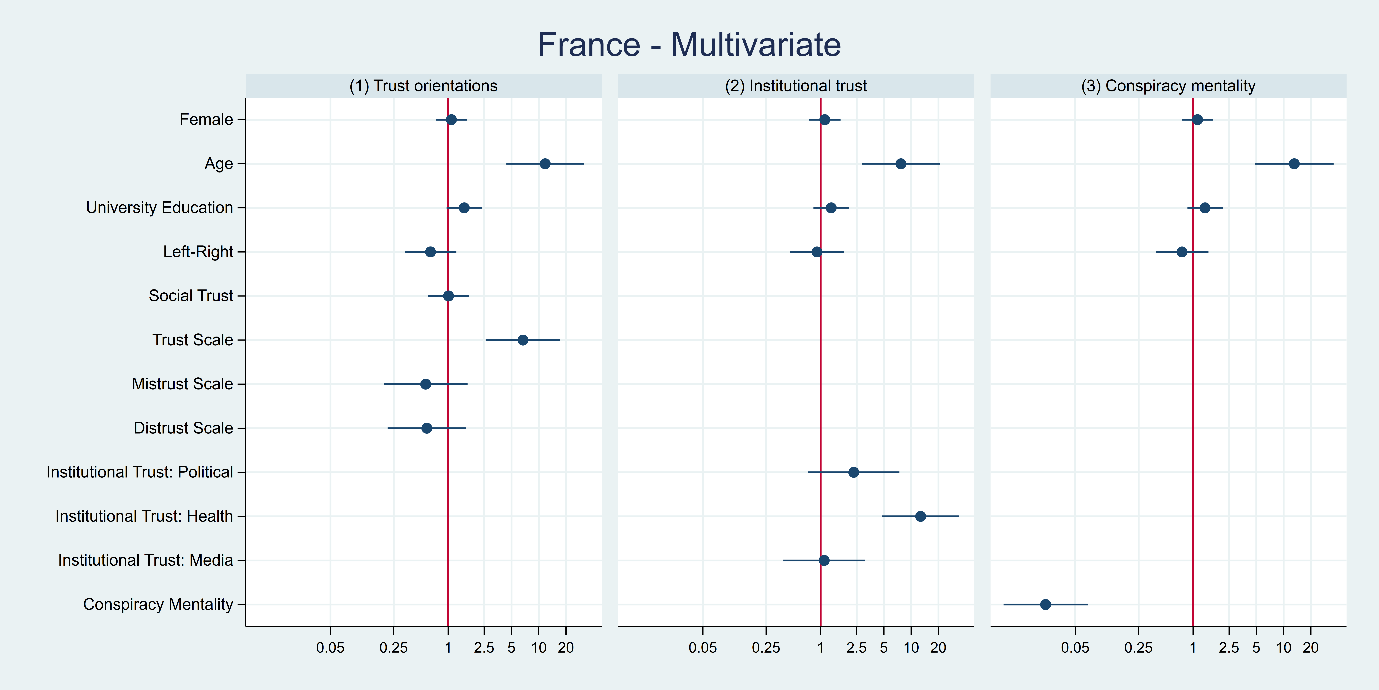


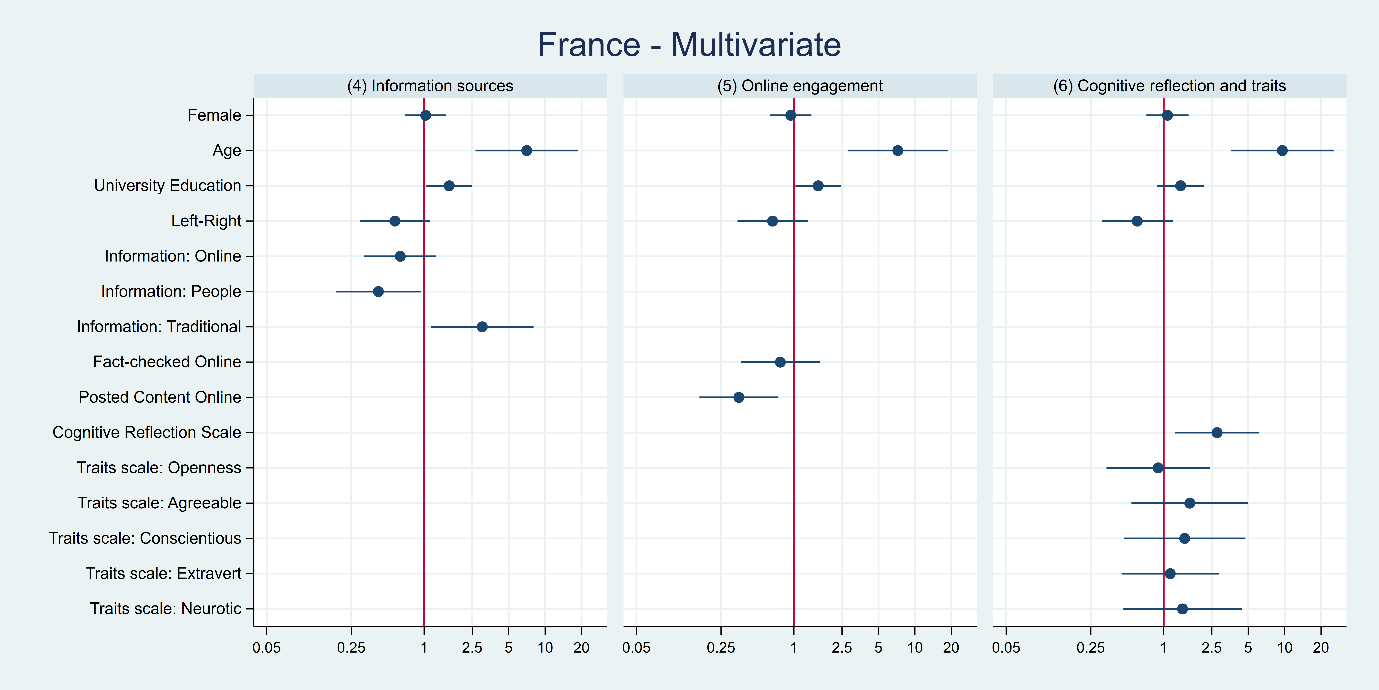


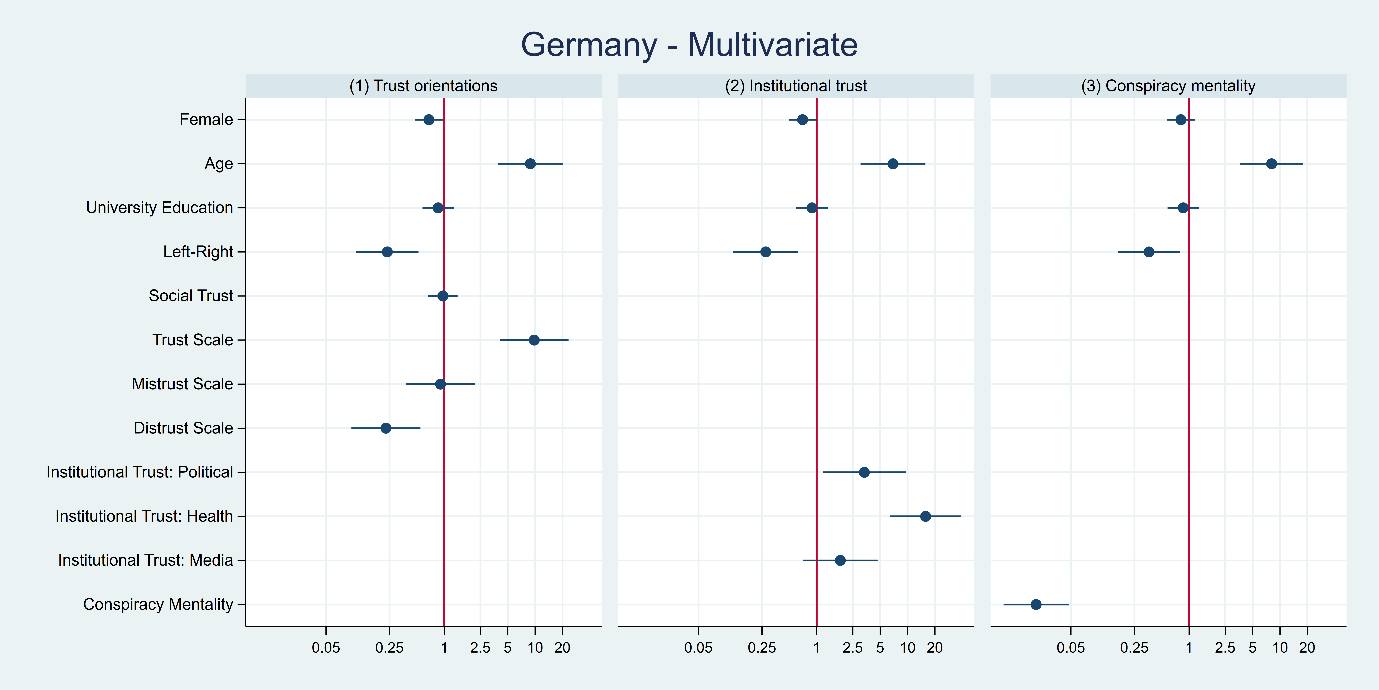


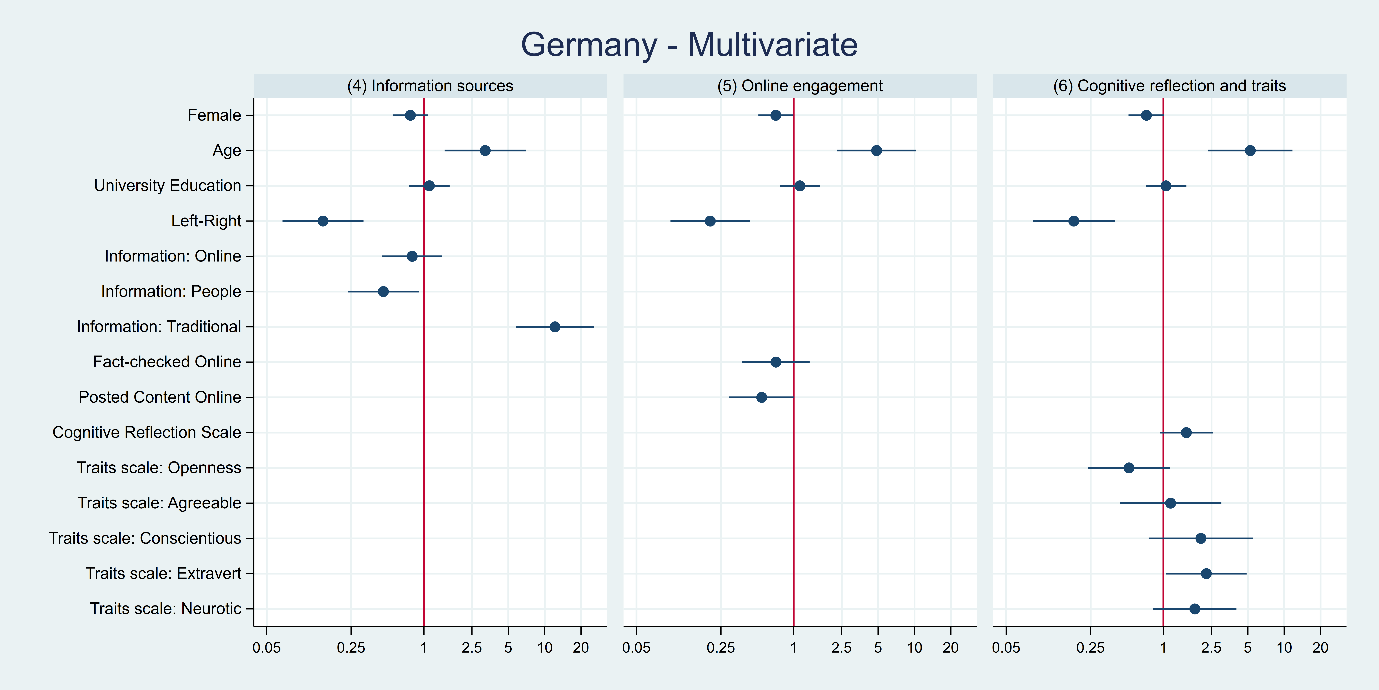


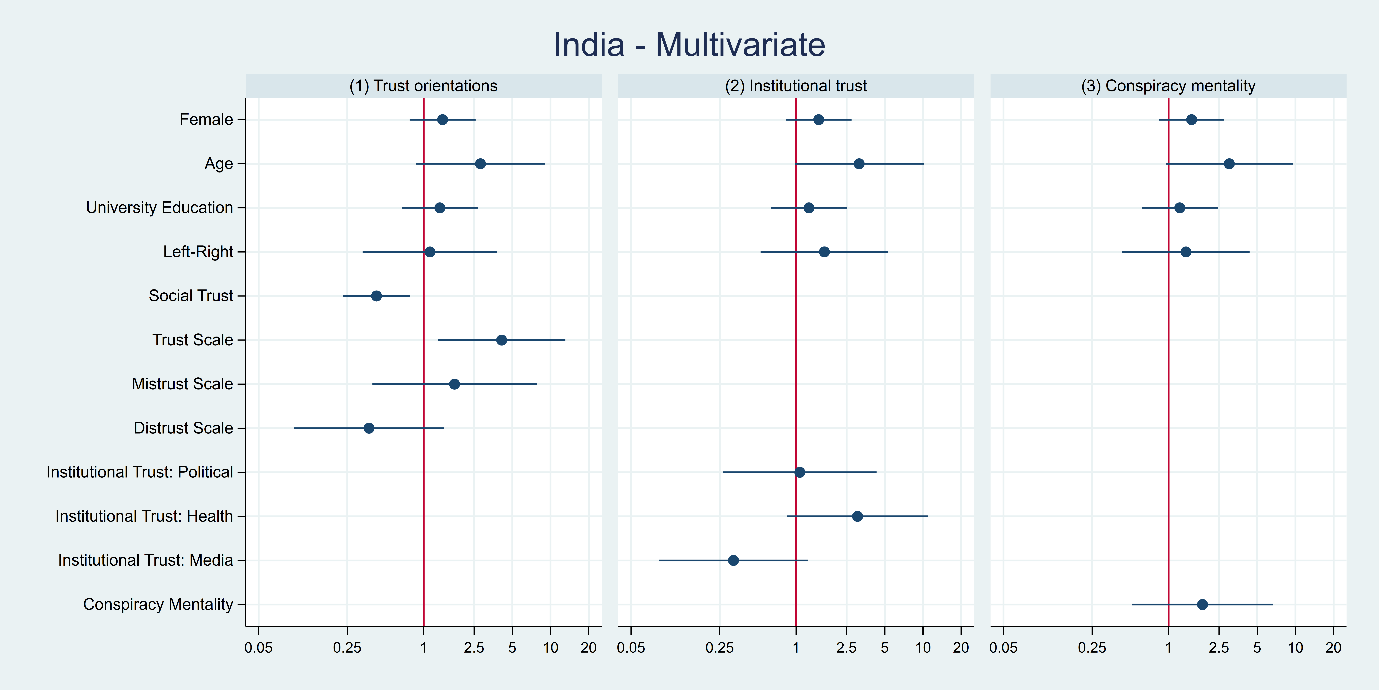


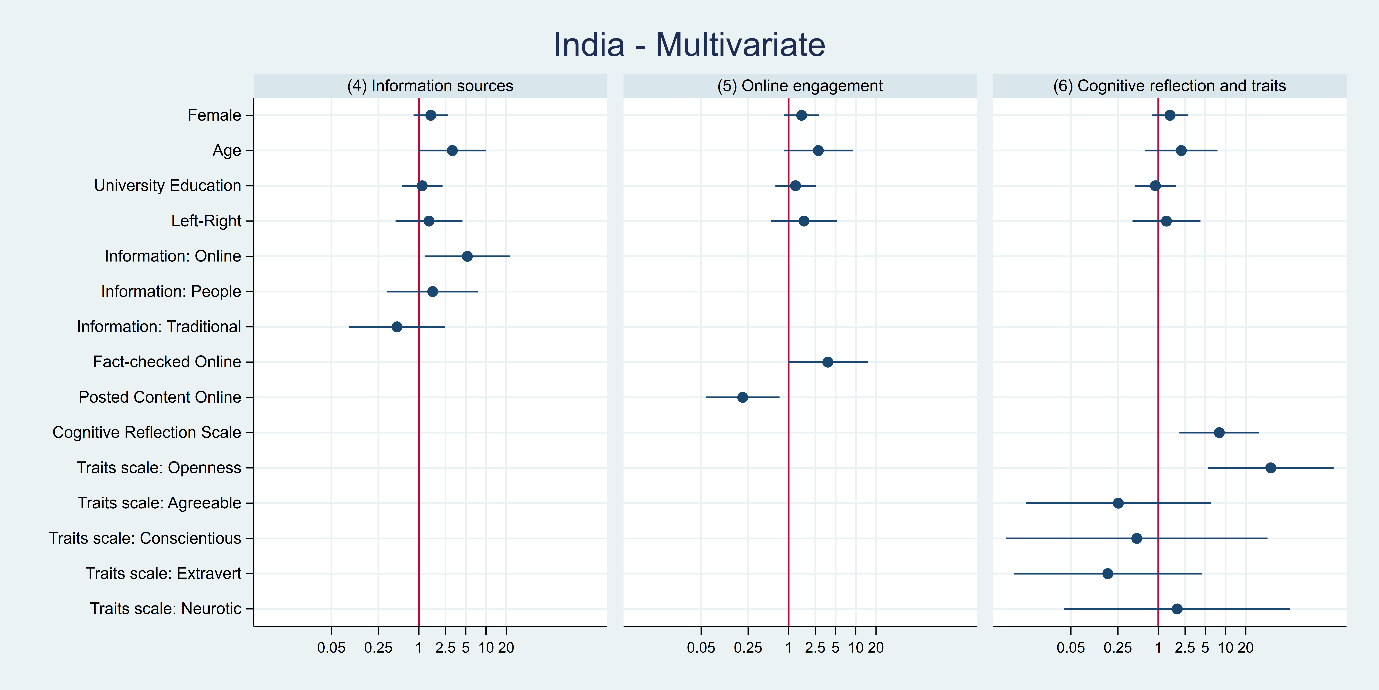


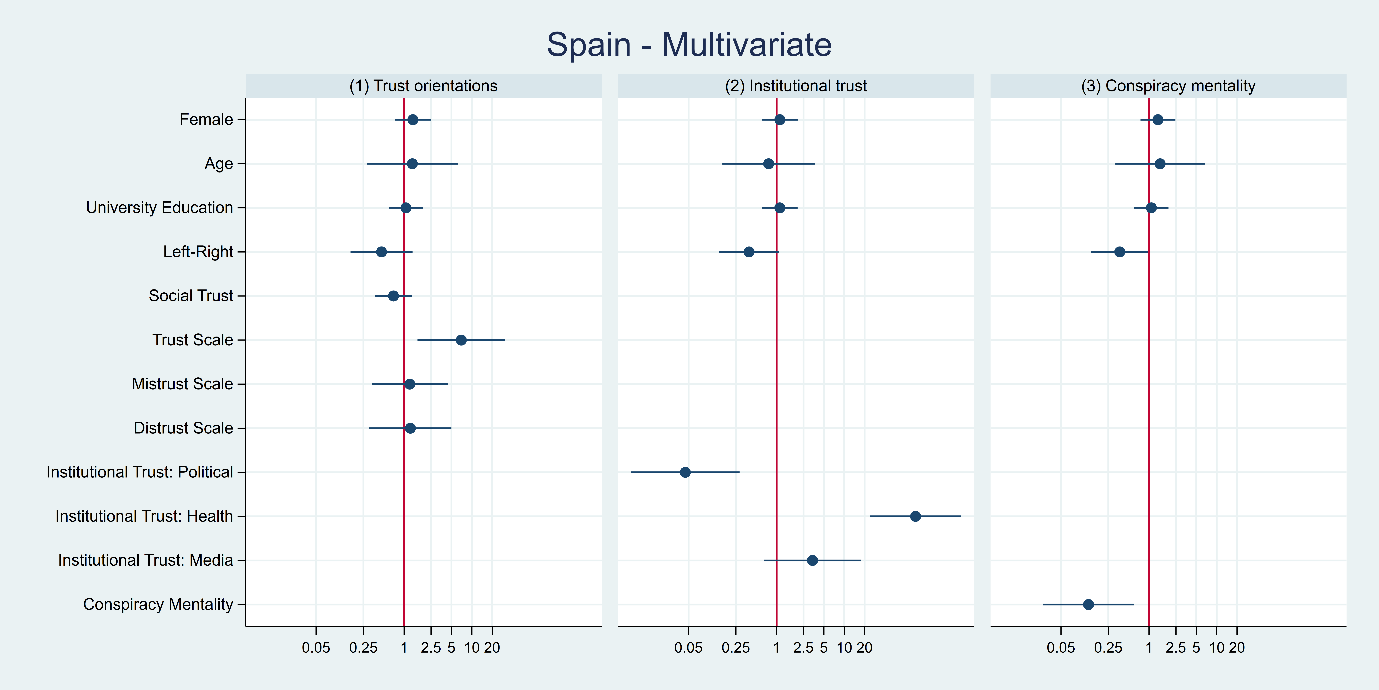


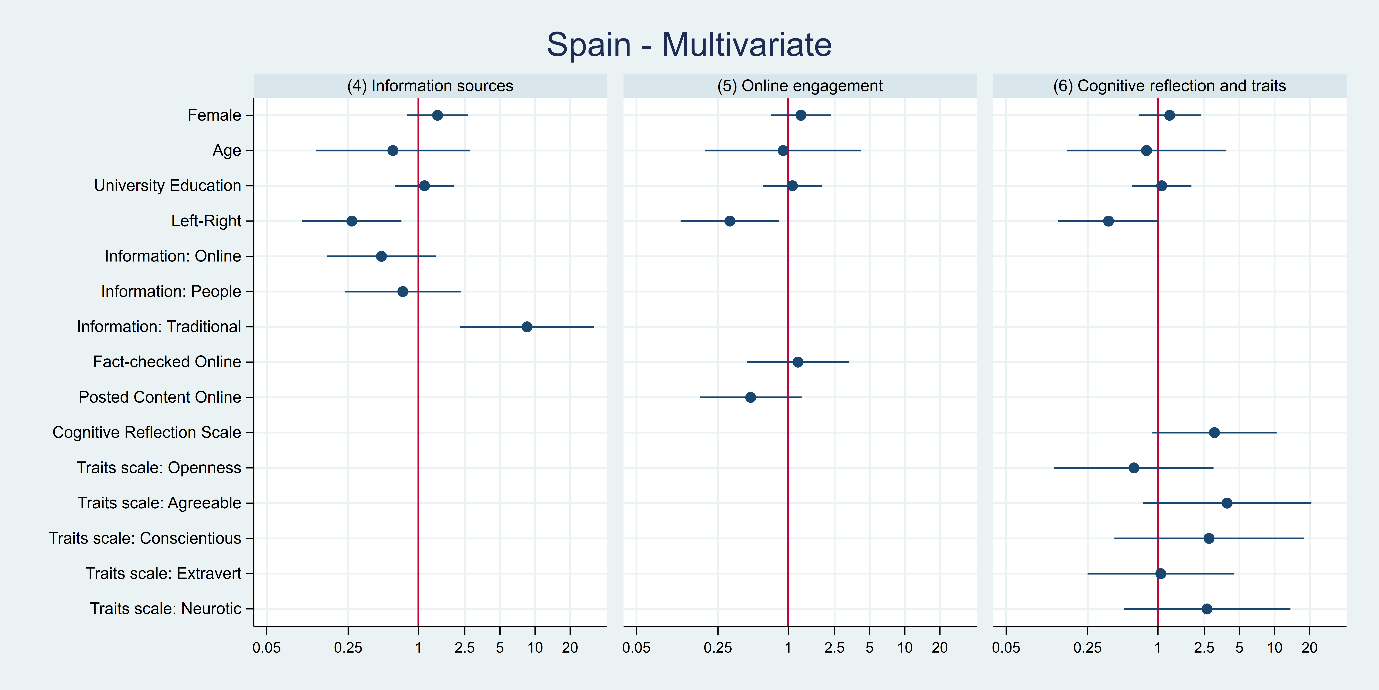

Supplement: Supplementary data 1 [file mmc1.docx]
